# Optoacoustic Imaging and Tomography: Reconstruction Approaches and Outstanding Challenges in Image Performance and Quantification

**DOI:** 10.3390/s130607345

**Published:** 2013-06-04

**Authors:** Christian Lutzweiler, Daniel Razansky

**Affiliations:** Institute for Biological and Medical Imaging, Technical University of Munich and Helmholtz Center Munich, Ingolstadter Landstraße 1, Neuherberg 85764, Germany; E-Mail: christian.lutzweiler@helmholtz-muenchen.de

**Keywords:** optoacoustic imaging, photoacoustic tomography, image reconstruction, quantification, multispectral optoacoustic tomography, inverse problem, light transport, spectroscopic imaging

## Abstract

This paper comprehensively reviews the emerging topic of optoacoustic imaging from the image reconstruction and quantification perspective. Optoacoustic imaging combines highly attractive features, including rich contrast and high versatility in sensing diverse biological targets, excellent spatial resolution not compromised by light scattering, and relatively low cost of implementation. Yet, living objects present a complex target for optoacoustic imaging due to the presence of a highly heterogeneous tissue background in the form of strong spatial variations of scattering and absorption. Extracting quantified information on the actual distribution of tissue chromophores and other biomarkers constitutes therefore a challenging problem. Image quantification is further compromised by some frequently-used approximated inversion formulae. In this review, the currently available optoacoustic image reconstruction and quantification approaches are assessed, including back-projection and model-based inversion algorithms, sparse signal representation, wavelet-based approaches, methods for reduction of acoustic artifacts as well as multi-spectral methods for visualization of tissue bio-markers. Applicability of the different methodologies is further analyzed in the context of real-life performance in small animal and clinical *in-vivo* imaging scenarios.

## 1. Introduction

Nowadays, the terms *optoacoustic* and *photoacoustic* are equally used to describe the effect of acoustic wave generation by transient light absorption. Optoacoustic sensing and imaging draws its roots from the discovery of the *photophone* by Bell and his assistant Tainter in 1880, which the inventors used as the first practical wireless telephony or, in fact, optical communication device [1]. Bell also named the effect the *photophonic phenomenon* [2] and later suggested the so-called *spectrophone* [3]. Following its discovery in solid and liquid media, the effect had been further confirmed by Tyndall and Rontgen to occur in gases [4,5]. However, despite multiple early attempts to put the photophone into practical use, mainly for military communications [6], it was not until 1938 that Veingerov developed the first widely accepted implementation of the phenomenon, which he called method for gas analysis based on Tyndall-Rontgen *optic-acoustic* effect [7,8]. The term *photoacoustic* was later adopted in 1970s when sensitive spectroscopy of solids was first demonstrated [9,10]. Rosencwaig was the first to suggest the phenomenon for biological sensing [11]. However, due to the lack of appropriate laser sources and ultrasound detection technologies, theoretical understanding of the underlying phenomena as well as backlogs with the algorithmic and data processing capacities, utilization of optoacoustics for imaging of real biological tissues and organisms has only evolved in the last decade [12–14].

The field of biological optoacoustic imaging is developing tremendously with new technical approaches and applications continuously emerging. By combining now commercially available pulsed laser technology in the nano-second range and sensitive acoustic detectors, it was shown possible to generate opto-acoustic responses from tissue that carry significant spatially-resolved biochemical information [15]. Due to its hybrid nature, *i.e.*, optical excitation and acoustic detection, the optoacoustic imaging technology benefits both from the rich and versatile optical contrast and high (diffraction-limited) spatial resolution associated with low-scattering nature of ultrasonic wave propagation as compared to photon propagation [16].

In vascularized tissues, highly absorbing hemoglobin manifests good optoacoustic contrast. It is therefore natural for optoacoustics to attain high fidelity images of vascular anatomy, dynamic microcirculation, tumor neovascularization, as well as blood oxygenation levels deep within highly diffuse tissues without introduction of contrast agents [17,18]. Besides blood-related contrast, optoacoustics is sensitive to other intrinsic tissue contrast [19]. Nevertheless, imaging of extrinsic photo-absorbing agents may offer important contrast advantage and imaging specificity but would typically require differentiation of these agents on top of spectrally varying background tissue absorption. In response, the so-called multi-spectral optoacoustic tomography (MSOT) technique relies on the spectral identification of chromophoric molecules and particles distributed in tissue over background tissue absorption [15,20]. Pulses of different wavelengths are used, in a time-shared fashion, whereas the wavelengths are selected to sample a certain spectral characteristic in the absorption spectrum of intrinsic bio-markers and reporter agents of interest. Essentially, when operated at multiple wavelengths, optoacoustics is capable of resolving spectral signatures of chromophores in three dimensions (volumetrically), *i.e.*, it can sense color, or more precisely, listen to color.

Despite its great promise, like ultrasound, optoacoustic imaging possesses several limitations related to imaging through acoustically mismatched areas, such as lungs and bones. Furthermore, even though spatial resolution performance here does not directly suffer from light scattering in tissue, it is still affected by penetration limits and signal degradation due to light attenuation. Therefore, whole-body imaging with optoacoustics is currently only possible in small animals whereas clinical applications are limited to relatively superficial, low attenuating or otherwise conveniently accessible areas of the body. Optoacoustic imaging is indeed a rapidly evolving area in biomedical imaging sciences. Its *in-vivo* use puts forth a number of challenging problems demanding intensive investigations, from imaging instrumentation, quantified reconstruction algorithms, spectral processing schemes, detection sensitivity, and other technical issues, to biology-related topics, such as effectiveness of imaging contrast approaches or animal handling. We refer an interested reader to some of the recently published review articles summarizing on progress of optoacoustic imaging applications [15,21,22]. On the technical side, while some focused reviews address the mathematical inverse acoustic problem [23] and quantification challenges of multispectral optoacoustic reconstruction methods [24], the current paper attempts to comprehensively cover the most recent experimentally-driven algorithmic developments in the optoacoustic field and also review some newly introduced image acquisition methodologies and selected practical *in vivo* imaging approaches.

## 2. Principles of Optoacoustic Imaging

### 2.1. The Optoacoustic (OA) Effect

**Figure 1.**
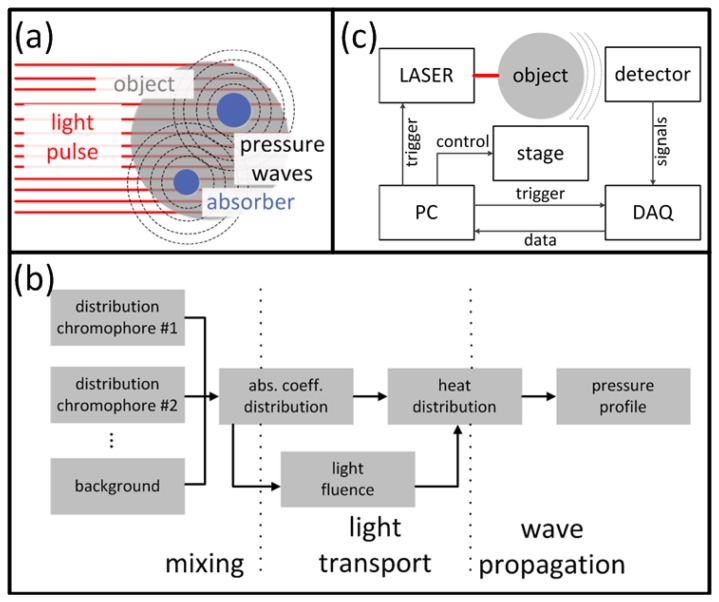
**(a)** Illustration of the optoacoustic effect. The object is illuminated with a light pulse and photons are absorbed within the object. The optical energy is converted to mechanical energy and gives rise to propagating pressure waves; (**b**) Schematic of the main physical processes involved in optoacoustic imaging: The distributions of different chromophores contribute to optical absorption and they mix to the spatially varying absorption distribution. The transport of light determines the local heat distribution by both absorption distribution and light fluence. The deposited heat excites pressure waves which propagate to the detectors' locations according to acoustic wave equation; (**c**) Schematic of a typical setup for optoacoustic imaging: A laser illuminates the object. The pressure waves are captured by one or many detector/s and their signals are digitized by a data acquisition system (DAQ). A stage moves the object relative to the detector for capturing multiple projections. A PC controls the imaging process and stores the data.

Optoacoustic (OA) imaging is based on absorption of light radiation in tissue and conversion of the deposited energy into heat (Figure 1(a)). This results in an expansion of the tissue and mechanical stress which propagates in the form of pressure waves. Typically, light in the visible or near-infrared spectrum (400 nm–1,200 nm) is used for excitation of OA responses due to the relatively weak absorption of biological tissues in this spectral region, also known as the *optical window*. Alternatively, tissue can be excited with energy in the radiofrequency and microwave spectra [25,26], which is however not included in the scope of this paper. A large variety of chromophores absorb at the optical wavelengths of interest, which further leads to a high contrast between different tissues with varying chromophore concentration. For biomedical applications, examples of usable contrast include intrinsic chromophores (e.g., hemoglobin in its oxygenated and deoxygenated form, melanin, fat), extrinsically administered agents (e.g., nanoparticles, fluorophores [27–30]) or genetically encoded markers, such as fluorescent proteins [31]. Vast majority of the modern OA imaging setups utilize lasers that emit ultra-short pulses, mainly due to a better signal to noise performance and parallelization capacity [32]. In this paper, we therefore limit our discussion to the theory and applications of pulsed OA sources, although the interested reader is indeed encouraged to explore more about OA imaging using modulated continuous-wave sources [33,34].

To simplify the model of the induced pressure variations, two assumptions are usually made on the duration *x* of the laser pulse: the first is the so-called thermal confinement assuming that the heat conductance during a laser pulse in one image voxel does not affect the neighboring voxels (*τ* ≪ Δ*l*/4*D_t_*, where Δ*l* is the dimension of the voxels and *D_t_* is the thermal diffusivity). The second is the so-called stress confinement assuming the volume expansion during the laser pulse to be negligible (*τ* ≪ Δ*l*/*c*, where *c* is the speed of sound). With these simplifications, the initially induced pressure *p*_0_(*r*) is generally proportional to the total absorbed optical energy density *H*(*r*):

(1) $$p_{0}(r)=p(r,t=0)=\Gamma \cdot H(r)\frac{\beta c^{2}}{C_{p}}\cdot H(r)$$

where Γ is the dimensionless Grüneisen parameter, *β* is the isobaric thermal expansion coefficient, and *C_p_* is the isobaric specific heat capacity. In a more general sense, Γ represents the combined OA efficiency of conversion from heat into pressure. In contrast to significant spatial variations in the absorption coefficient, Γ does not normally exhibit dramatic variations among different soft tissues, thus it is most conveniently assumed to be constant. Nevertheless, accounting for spatial variations in Γ may still benefit the efforts on quantification of optoacoustic images [35,36]. Moreover, some studies attempted utilizing the strong dependence of the Griineisen parameter on temperature in order to sense temperature optoacoustically [37,38].

For biomedical applications, it is the distribution of the absorption coefficient *μ_a_*(*r*) that is usually directly related to the concentration *c_i_*(*r*) of the chromophores rather than the totally absorbed energy *H*(*r*). Both are related via the expression:

(2) $$H(r)=\mu_{a}(r)\cdot \Phi (r,\mu_{a},\mu_{s},g)$$

where **Φ**(***r,μ_a_,μ_s_,g***) is the light fluence (or photon density), ***μ****_s_* is the scattering coefficient and ***g*** is the anisotropy factor. Although it might seem that ***H***(***r***) is a simple product, it depends nonlinearly on the absorption coefficient ***μ_a_*** because the light fluence generally depends on the underlying optical properties, among them ***μ_a_*** itself. Thus, it is important for image quantification purposes to account for the light fluence since it may considerably vary as a function of depth. Figure 1(b) summarizes the main contributions to the OA signal generation, which need to be accounted for when developing accurate image reconstruction algorithms.

### 2.2. The Optoacoustic Wave Equation

The initial goal of optoacoustic imaging is to retrieve the initial pressure distribution inside the object due to the absorbed laser energy. However, the generated pressure fields can normally only be measured outside the object. Propagation of pressure toward the detection point is described by the optoacoustic wave equation, which, for a non-absorbing homogeneous medium, is written as [39]:

(3) $$\nabla^{2}p(r,t)-\frac{1}{c^{2}}\frac{\partial^{2}}{\partial t^{2}}p(r,t)=-\frac{\beta }{C_{p}}H(r)\cdot \delta \prime (t)$$

**Figure 2.**
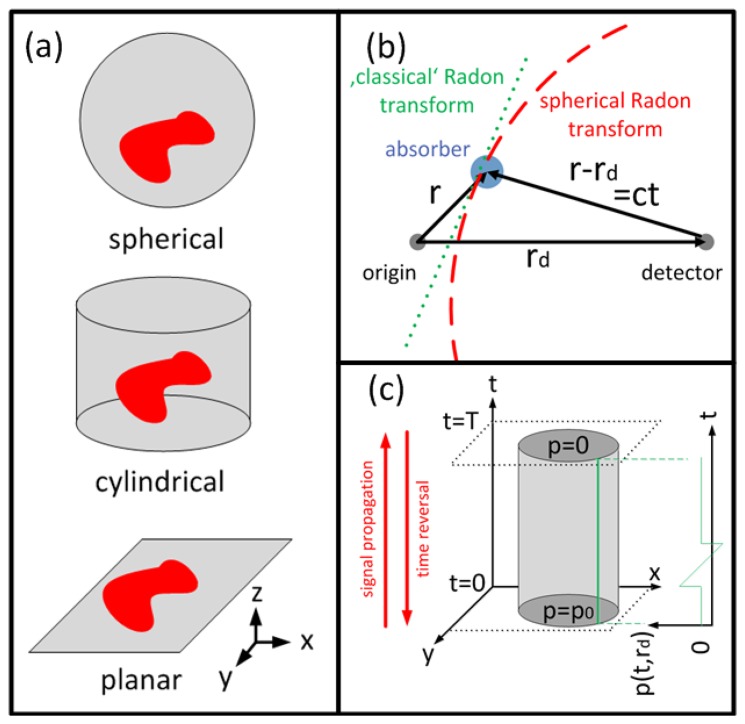
**(a)** Most common OA geometries with detectors located on a spherical, cylindrical or planar surface; (**b**) Schematic of the spherical Radon transform: The detector integrates over the pressure from locations with the same time-of-flight *t*. The pressure *p_d_*(*r_d_*, *t*) is the sum over a sphere with radius *c* · *t*; (**c**) Illustration of the time reversal principle: The initial pressure *p*_0_(*r*) inside the volume propagates starting from *t* = 0 and is captured on the detection surface. It becomes zero inside the volume for *t* > *T*. In time reversal reconstructions one re-emits the pressure profile on the surface in time reversed order starting from zero initial conditions at *t* = *T* and propagates it backwards in time to result the initial pressure at *t* = 0.

For tomographic data acquisition, the detectors are placed on a (closed) surface *S* surrounding the volume of interest. The most common detection patterns are a spherical, a cylindrical and a planar surface or their 2-D counterparts (Figure 2(a)). On the surface the temporal pressure profile *p_d_*(*r_d_*, *t*), *r_d_* ∈ *S*, *t* > 0, is considered known. To reconstruct an OA image from *p_d_*(*r_d_*, *t*), the initial pressure *p*_0_(*r*) satisfying:

(4) $$\begin{array}{ll} \nabla^{2}p(r,t)-\frac{1}{c^{2}}\frac{\partial^{2}}{\partial t^{2}}p(r,t)=0 & r\in {\mathbb{R}}^{3},t\geq 0\\ p(r,t=0)=p_{0}(r) & \\ \frac{\partial }{\partial t}p(r,t=0)=0 & \\ p(r_{d},t)=p_{d}(r_{d},t) & r_{d}\in S,t>0 \end{array}$$

has to be found. To solve this partial differential equation, one usually takes advantage of the free-space Green's function *G*(*r*, *r*′, *t*, *t*′). It relates the pressure at two different spatio-temporal points, *i.e.*,

(5) $$G(r,r\prime ,t,t\prime )=\frac{\delta ((t-t\prime )-|r-r\prime |/c)}{\left|r-r\prime \right|}$$

Consequently, the pressure profile *p_d_*(*r_d_*, *t*) on the detection surface *S* can be directly related to the initial pressure *p*_0_(*r*) via a Poisson-type integral:

(6) $$p_{d}(r_{d},t)=\frac{1}{4\pi c}\frac{\partial }{\partial t}{\int dA\frac{p_{0}(r)}{\left|r_{d}-r\right|}|}_{ct=|r_{d}-r|}$$

This type of equation is known as spherical Radon transform or spherical mean transform. This is because contributions of all sources that are located on the surface of a sphere with radius *R* = *c* · *t* = |*r_d_* − *r*| from the detector are integrated (Figure 2(b)).

### 2.3. Imaging Instrumentation

**Figure 3.**
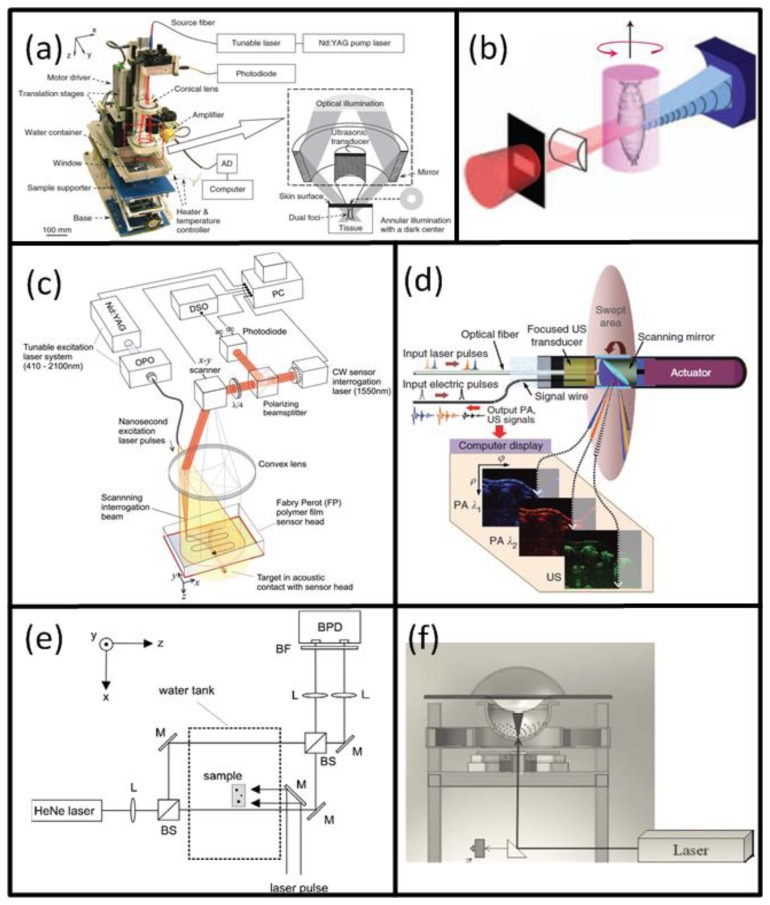
Illustration of different OA setups. (**a**) Schematic of the OA microscope with a ring-shaped illumination used by Zhang *et al*. for functional high resolution imaging [44]. *Reprinted with permission from Nature Publishing Group*; (**b**) Confocal selective-plane light sheet illumination and detection pattern for tomographic multispectral *in vivo* small animal imaging by Razansky *et al*. [45]. *Reprinted with permission from Nature Publishing Group*; (**c**) Illustration of the OA raster scanning system by Zhang *et al*. based on a Fabry-Perot interferometer [46]. *Reprinted with permission from Optical Society of America*; (**d**) Combined endoscopic system presented by Yang *et al*. for co-registered US and dual-wavelength functional OA images *in vivo* [47]. *Reprinted with permission from Nature Publishing Group*; (**e**) Schematic of the OA tomography system by Paltauf *et al*. with an integrating line detection based on a Mach-Zehnder interferometer [48]. *Reprinted with permission from Optical Society of America*; (**f**) Illustration of the three-dimensional OA tomography system by Kruger *et al*. for angiography of the breast based on 128 detectors located on a rotating hemisphere [49]. *Reprinted with permission from American Association of Physicists in Medicine*.

A typical optoacoustic setup consists of several key components, an example shown in Figure 1(c). In the pulsed excitation mode, the tissue is illuminated by a laser emitting monochromatic pulses of light with a typical duration of some nanoseconds. For deep tissue imaging applications, optical parametric oscillators are often used to provide a tunable wavelength in the spectrum of interest with pulse repetition rate in the order of a few tens of Hertz and per-pulse energies in the millijoule range. In optoacoustic microscopy and other superficial applications, where such high per-pulse energies are not required, other types of sources in the microjoule and nanojoule range are considered as well, including high repetition passive Q-switched and dye lasers [40,41], laser diodes [42] and fiber lasers [43]. For tomographic imaging, the optoacoustically-generated pressure profiles are subsequently captured with detectors surrounding the object whereas the acoustic coupling between object and detector is usually ensured by water or gel medium. In contrast to ultrasonic (US) imaging, OA signal amplitudes are relatively low while their spectral content is broad, spanning the frequencies from several tens of kHz up to a hundred MHz for micron-scale structures. Thus, high sensitivity, ultrawide bandwidth as well as good tomographic coverage are key for ensuring image quality. In addition, for acquisition of spatially resolved data with any type of detector, either a single detector is scanned around the object or, alternatively, multiple detection elements acquire data in parallel. The latter allows fast data acquisition, e.g., for reconstruction of images from a single laser shot with commercially available or tailored detection arrays. Figure 3 illustrates the broad variety of OA setups presented in the literature [44–49] including OA raster scanning (Figure 3(a,c)), tomography (Figure 3(b,e,f)) and endoscopy systems Figure 3(d). The signals were detected optically with an interferometer for the setups in Figure 3(c,e), whereas in the other systems conventional piezoelectric detection elements were used.

Two main categories of sensors are used to detect OA signals: The most commonly used type is based on piezoelectric elements [50,51], such as piezocomposite- or polymer-based films, which are also widely utilized in US imaging. This well developed technology possesses relatively low cost of implementation and can be readily applied for highly sensitive measurements. However their sensitivity scales with detector's size, making miniaturization challenging. Of the second type are optical resonators and interferometry detectors like microring resonators [52,53], Fabry-Perot interferometers [46], Mach-Zehnder interferometers [48], or fiber Bragg gratings [54], which are sensitive to changes in the length of the optical path induced by pressure waves. Interferometric detectors usually possess ultrawideband detection characteristics and further allow all-optical optoacoustic imaging, in some cases also greatly simplifying delivery of the excitation light to the imaged object through transparent optical components. Sensitivity of interferometric detectors is not directly dependent upon the physical size of the detection element, which simplifies miniaturized designs suitable for small scale imaging, such as intravascular or endoscopic applications. On the other hand, interferometric detection approaches are usually difficult to parallelize in order to achieve faster tomography while it is also not always possible to increase detection sensitivity by integration of signals over large areas. Recently, other detection technologies have been introduced and successfully applied for optoacoustic imaging, such as capacitive micromachined ultrasonic transducers (CMUT) [55–57]. The advantages and disadvantages of the various optoacoustic sensor approaches are summarized in Table 1.

Following their detection, the optoacoustic waveforms are pre-amplified and digitized by a data acquisition system (DAQ). For tomographic data acquisition, a motion stage can be translated or rotated in order to alter the position of the detector/s with respect to the imaged object or vice versa in order to increase the amount of the available data points (projections) around the imaged area. A sufficiently high number of projections and a suitable detection pattern are crucial for good image quality. A PC controls the acquisition process, stores the data and is used to reconstruct and render the images. Prior to image formation, an additional signal processing step normally takes place that may include some of the following: averaging over multiple pulses to enhance signal-to-noise ratio (SNR), denoising the signals [58,59], deconvolving the signals from electrical frequency response of the detector [60,61], band pass filtering to remove noise and/or enhance visibility of certain spatial frequency components in the images.

## 3. Inversion of the Optoacoustic Wave Equation

The first step to obtain OA images consists in reconstructing the initial pressure distribution, *i.e.*, solving Equation (4) or inverting Equation (6). Since the early reconstruction approaches were introduced [39,62,63], plenty of research has been done on the problem in the last decade, driven from both theoretical interest and the need to improve images acquired from specific experimental setups.

### 3.1. Types of Inversion Algorithms

The inversion schemes are usually based on idealized assumptions of infinitely small point detectors located on a closed measurement surface and a non-absorbing homogeneous acoustic medium. Modifications for deviations from these idealized assumptions will be further presented below.

An inversion formula was initially presented by Norton *et al*. for ultrasonic imaging [62]. The basic idea is to represent the solution *p*_0_(*r*) as a convergent series:

(7) $$p_{0}(r)=\sum_{k}\alpha_{k}\cdot \Psi_{k}(r)$$

where Ψ*_k_*(*r*) are eigenfuctions of the negative Laplacian −Δ in the domain of the measurement geometry and *α_k_* are the so-called Fourier coefficients. The coefficients *α_k_* can be calculated from *pd*(*r_d_*, *t*). Although it has been shown that series solutions exist for arbitrary closed detection surfaces [64], only the geometries with known eigenfunctions (sphere, cylinder, plane) are usually of practical experimental interest [63–67].

For a planar detection geometry, this approach results in a fairly simple reconstruction algorithm. First, the signals *p_d_*(*x*, *y*, *t*) are transformed to Fourier space in *x*, *y* and *t* coordinates, resulting in *p_d_*(*k_x_*, *k_y_*, *ω*). Second, the temporal component is mapped to its corresponding spatial coordinate with the dispersion relation $\omega^{2}=c^{2}(k_{x}^{2}+k_{y}^{2}+k_{z}^{2})$, resulting in *p*_0_(*k_x_*, *k_y_*, *k_z_*). Finally, the reconstruction is obtained by applying the inverse Fourier transform in all three spatial coordinates. In this way, fast reconstructions of 3D volumes can be obtained using an FFT algorithm. A similar FFT-based approach has been presented by Kunyansky [64] for a finite cube used as the detection surface instead of an infinite planar surface which has to be truncated in practical cases. For a spherical measurement geometry, Wang *et al*. have recently derived a simple FFT-based algorithm in the frequency domain [68].

Another approach is based on closed-form analytical filtered back-projection (BP) formulae in the time domain [69–73]. This type of formula is commonly used in computed x-ray tomography to invert the classical Radon transform. Similar to Equation (7), the initial pressure is obtained by integrating the detected signals over spherical surfaces. Prior to or following the integration, a filter function is applied. The first exact back-projection formulae were obtained by Finch *et al*. for a sphere in 3-D [69] and later also in 2-D [70]. Xu *et al*. presented an approximate BP formula for a spherical detection geometry [72], which was later generalized to the widely used universal back-projection (UBP) formula [73]:

(8) $$p_{0}(r)=\frac{1}{4\pi c^{2}}{\int dS\frac{1}{t}\left[\frac{p_{d}(r_{d},t)}{t}-\frac{\partial p_{d}(r_{d},t)}{\partial t}\right]|}_{ct=|r_{d}-r|}$$

The latter scheme can be efficiently applied to spherical, cylindrical or planar (with 4*π* replaced by 2*π*) geometries. In the far-field approximation with detectors located far from the object, only the derivative term in Equation (8) contributes significantly.

Time reversal inverse methods are based instead on the Huygens' principle [74–76]: In three dimensions, the waves from an initial pressure distribution *p*_0_(*r*), confined inside a finite volume *V*, will leave the volume after a finite duration *T* while the pressure *p*(*r*, *t*) inside the volume will vanish for *t* > *T*. Although Huygens' Principle does not hold in two dimensions, the pressure inside the volume usually decays fast enough to result in good reconstructions for large *T*. Time reversal methods aim in solving Equation (4) backwards in time: Starting from zero initial condition at time *t* = *T*, the pressure distribution is propagated backwards in time step-by-step, reemitting the pressure profile *p_d_*(*r_d_*, *t*) on the detection surface for each time point (Figure 2(c)). Arriving at *t* = 0, the pressure distribution *p*(*r*, *t*) equals the sought-after initial pressure *p*_0_(*r*). Time reversal methods can be implemented by finite differences methods [75] or by k-space methods [77], which accelerate inversion by using larger time steps, and furthermore offer greater flexibility as they generally work for arbitrary closed measurement surfaces.

Whereas series solution or the back-projection formulae perform an approximated inversion of Equation (6) analytically, the so-called model-based (MB) algorithms seek instead for an accurate numerical solution [78–80]. Rosenthal *et al*. presented an algorithm that is based on a discretized semi-analytical forward model *M̃* of the wave propagation that linearly maps the discretized initial pressure *p̃*_0_ to the corresponding discretized signals *p̃_d_* [79]. Numerical models can also be obtained by finite element (FE) calculations of Equation (4), which can be implemented in the time or frequency domain [81–83]. The image is then reconstructed by finding a solution that minimizes the difference between the measured data *p̃_d_* and the pressure corresponding to the forward-modeled image *p̃*_0_:

(9) $${\tilde{p}}_{d}=\underset{{\tilde{p}}_{0}}{\text{argmin}}{\left\|\tilde{M}{\tilde{p}}_{0}-{\tilde{p}}_{d}\right\|}_{2}+P({\tilde{p}}_{0})$$

In Equation (9), *P* is an optional regularization term, such as Tikhonov or total variation regularization, which is intended for stabilization of the numerical inversion. The solution can be calculated via the Moore-Penrose pseudo-inverse *M̃*^†^ [84]. Although calculation of *M̃*^†^ may become burdensome in terms of CPU time and memory consumption, it needs to be calculated only once for a given experimental system. Then the reconstruction process reduces to a simple and fast matrix-vector multiplication:

(10) $${\tilde{p}}_{0}={\tilde{M}}^{†}{\tilde{p}}_{d}$$

Alternatively, the solutions can be calculated iteratively, for example with the LSQR algorithm [85] that takes advantage of the sparse nature of the model matrix. Benefiting from the advances in computation technology, MB algorithms have drawn considerable interest in the recent years because of their flexibility to account for a broad variety of experimental imperfections in the model. However, their drawbacks are often associated with longer calculation times or a considerable consumption of memory needed to store the model (or its pseudo-inverse), especially when considering the full three dimensional problem [82].

### 3.2. Computational Efficiency

Besides image quality and technical feasibility, computational efficiency is yet another important criterion determining applicability of the different methodologies, especially when dealing with large 3D datasets [86] or in applications involving real-time rendering of images. For instance, different approaches can be characterized by their algorithmic complexity in reconstructing 3-D datasets-*O*(*n*^4^) for series solutions, *O*(*n*^5^) for BP algorithms, *O*(*n*^4^) for time reversal methods and *O*(*n*^9^) for calculating the pseudo-inverse in MB algorithms. The series solutions can result in particularly fast algorithms whenever fast methods for summation of the eigenfunctions are applicable (e.g., FFT), resulting in low complexity in the order of *O*(*n*^3^ · log*n*) [63,64,67]. In addition to the algorithmic complexity, additional implementation aspects include the different memory consumption requirements between algorithms that only involve standard linear algebra operations for inversion, such as BP, *versus* the extensive memory size needed to store matrices for some MB schemes. The graphic processing units (GPUs), which are increasingly used in medical imaging in the recent years due to their vast computational power [87,88], are expected to mitigate or significantly reduce complications concerned with computational complexities in optoacoustic imaging.

As mentioned above, MB algorithms offer better reconstruction accuracy and flexibility in accounting various experimental imperfections; however they demand large computational resources. Consequently, methods to overcome this problem have been presented. Wang *et al*. proposed a method that circumvents storing the large model matrix by approximating voxels in the image by means of spheres, whose corresponding pressure profiles are known analytically [80]. For the GPU implementation of their algorithm they reported a runtime of 313 min per iteration for a 3-D high resolution data set, whereas the corresponding BP reconstruction took only 46 s [87]. Rosenthal *et al*. developed an efficient framework for model-based inversions based on wavelet packets [89]. In this case, both the image and the signals are decomposed into a two-level wavelet-packet basis. The model matrix is approximately block-diagonal in this basis and thus an approximate inverse matrix can be calculated (and stored) easily. From the obtained inverse, the image is directly calculated in a more efficient and faster manner and can be then further improved iteratively. The authors reported a runtime of 0.1 s for a 2-D data set by direct reconstruction from the approximate inverse, which constituted a 40-fold increase in the reconstruction speed as compared to the regular iterative MB algorithm.

### 3.3. Reconstructions with Finite-Sized Detection Elements

**Figure 4.**
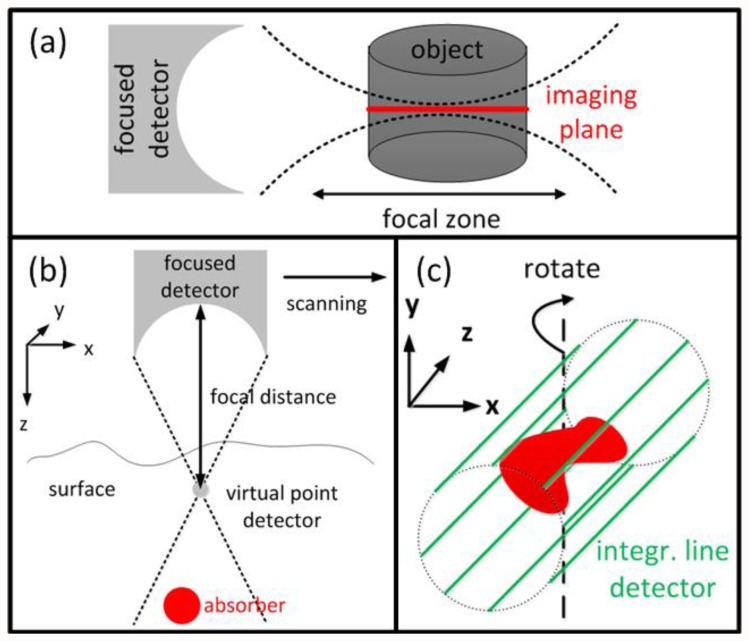
**(a)** Selective-plane detection scheme: The object is placed in the focal zone of a focused detector. Only the signals arising from a narrow region around the imaging plane are detected. Thus the reconstruction is reduced to two dimensions; (**b**) Illustration of the virtual detector concept: Signals measured with a large focused detector are assumed to be detected by a virtual point detector in the focal point with the signals delayed by a time according to the focal distance; (**c**) Schematic of an integrating line detector setup: The line detectors detect the signals of the object integrated in z-dimension. A complete set of data is obtained by rotation of the object relative to the detectors.

Due to mathematical and numerical simplicity, most inversion schemes assume infinitely small point detectors. Conversely, in experimental reality, larger detector sizes are often preferable as they lead to a better detection SNR, which in many cases scales with detector size. Furthermore, large area detectors are also required for focused detection geometries. For instance, if the object is placed in the focal zone of a cylindrically focused detector (Figure 4(a)), the signals acquired by the detector originate only from a small region close to the imaging plane. The reconstruction is thus effectively reduced to a 2-D problem. So data acquisition and image rendering can be performed faster or even in real-time [90–92]. OA microscopy setups often use spherically focused detectors which are in first approximation only collecting signals from a line, in which case images can be formed without complicated reconstruction algorithms. However, simplified assumptions readily lead to out-of-plane (out-of-focus) artifacts in the images, loss of quantification, highly anisotropic resolution and degradation of the overall image quality.

Li *et al*. introduced the virtual detector concept to OA [93–95] in order to improve the reconstructions from focused detectors outside the focal point (Figure 4(b)). The detected signals are assumed to originate from a virtual point detector located in the focal spot, delayed by the time corresponding to the focal distance. These signals can then be used to form an image, e.g., by a simple delay-and-sum algorithm for OA microscopy or a back-projection algorithm for tomographic reconstructions.

A very straightforward reconstruction approach can be applied for flat detectors with dimensions much larger than the object (in contrary to point detectors with dimensions much smaller than the object). The so-called integrating line detectors (Figure 4(c)) simultaneously record signals along a line thus effectively the pressure field of the object integrated along a certain axis [96,97]. A complete tomographic data set is subsequently obtained by rotating the detectors' orientation. The reconstruction process is then performed in two steps: First, the integrated 2-D images are reconstructed for a given detector orientation with an arbitrary 2-D method. In a second step, the 3-D reconstruction is obtained from the integrated 2-D reconstructions for all detector orientations with the inverse Radon transform. Alternatively to line detectors, detection elements, that are integrating in two dimensions or integrating in one and focused in the second dimension, have been suggested as well [98,99].

An interesting approach, which is also based on detection of integrated pressure fields, was presented by Nuster *et al*. [100]. Instead of detecting a time-resolved integrated field along a certain line, the approach consists of recording the full field at a given time instant *t_d_* using a CCD camera. In this way, the integrated acoustic pressure field $p_{d}^{\left(z\right)}(r_{\left(x,y\right)},t_{d})$ is detected in a direction perpendicular to the imaging plane. *t_d_* is chosen in such a way that essential parts of the waves have already left the reconstruction domain. A volumetric image can be then reconstructed from a few simple steps: (1) The 2-D pressure field is integrated (Radon transform) along a fixed direction in the imaging plane. (2) The integrated 1-D signals are back-propagated in time from *t* = *t_d_* to *t* = 0 using D'Alembert's formula, which results an integrated 1-D image. (3) An integrated 2-D image is obtained by the inverse Radon transform from the integrated 1-D images for all in-plane directions. (4) A final 3-D image is reconstructed by the inverse Radon transform from the 2-D images for all orientations of the imaging plane.

Rosenthal *et al*. presented a generalized method that allows accounting for arbitrary-shape detectors [101]. It is based on including the effects of the exact detector shape in the forward model of a model-based inversion approach by temporal convolution. As a result, image artifacts related to finite detector size can be readily eliminated using this universal approach, while also improving spatial resolution of the images.

### 3.4. Effects of Heterogeneous Acoustic Properties

In real tissues, acoustic properties may significantly deviate from the idealized homogeneous properties initially assumed in the governing equations, which may lead to deterioration of the image quality. Indeed, speed of sound *c* may vary considerably for different types of tissue within a range of 1,400–1,600 m/s and be also spatially dependent (*c* = *c*(*r*)) [102]. Information on the speed of sound within the object can be obtained using various approaches, e.g., from *a priori* knowledge on the object, ultrasound or hybrid measurements [103,104], auto-focusing approaches [105] or can be reconstructed from the signals by a combined FE approach [106]. Although simplified series solutions exist in principle for optoacoustic reconstructions with variable speed of sound [107], iterative and time reversal methods were further shown efficient for this purpose [75,77,108].

An additional effect taking place is attenuation of the optoacoustic waves as they propagate through the tissue. Often these effects are also dispersive, *i.e.*, acoustic attenuation increases with frequency, which is usually modeled by exponential attenuation with a power-law of *ω^n^*, 1 ≤ *n* ≤ 2 [102]. The penetration depth from which acoustic waves can be detected also limits the imaging depth. Since the maximum OA resolution is determined by the frequency content of the signals [109], the resolution thus scales with depth. Time-gain compensation applied in US imaging cannot be used for OA signals because they are generally broadband. Instead, the attenuation is accounted for by adding a dissipation term in the time domain for time reversal methods [76] or by a complex frequency-dependent dispersion relation in an iterative approach [110].

**Figure 5.**
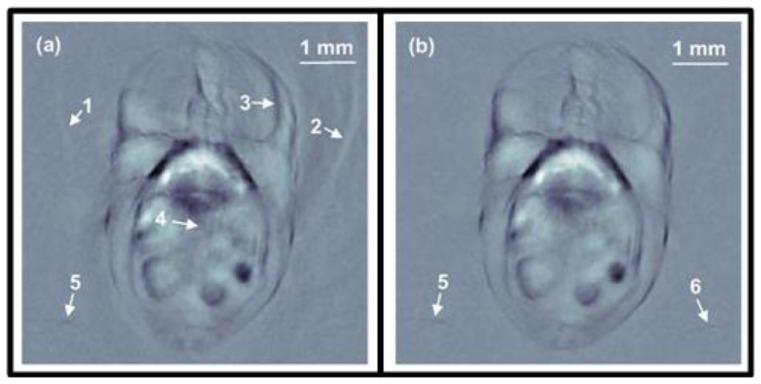
OA reconstruction of a zebra fish in the highly mismatching region of the swim bladder by Dean-Ben *et al*. [113]. (**a**) Reconstruction with universal back-projection algorithm (1–3: reconstruction artifacts; 4: smeared region near the liver; 5 + 6: pectoral fins); (**b**) Reconstruction with the statistically weighted back-projection algorithm accounting for the acoustic mismatch and reducing the image artifacts. *Reprinted with permission from IEEE*.

In media with heterogeneous acoustic properties, scattering and reflection of the acoustic waves may occur at boundaries of highly mismatching media. Examples include bones or air cavities such as the lung. Wang *et al*. studied the effects of reflection at soft and hard boundaries and were able to considerably improve image quality in the presence of reflections at planar boundaries [111], which might however not always be applicable to realistic imaging scenarios. Anastasio *et al*. showed that tomographic OA signals, acquired from multiple view angles (projections), contain complementary information thus images can be equally reconstructed from truncated signals, which may improve image quality in heterogeneous acoustic media due to data redundancy principles [112]. Dean-Ben *et al*. presented a statistically weighted BP algorithm that efficiently accounted for strong acoustic reflections [113,114]. The algorithm weights each detected signal by a factor that represents the probability of the signal being undisturbed during propagation. In this way, reconstruction artifacts arising from scattering can be suppressed (Figure 5).

### 3.5. Tomographic Reconstructions in Limited View Geometries

Ideal tomographic imaging scenarios assume that the object is fully surrounded by detectors on a closed surface, creating the so-called full-view scenario. Full view detection is favorable for the reconstruction process but cannot always be realized in experimental conditions, e.g., because the imaged region is simply not accessible for efficient detection from all directions or cannot be fully immersed in water for efficient acoustic coupling to the detectors. Such scenarios are known as limited view reconstructions and the inversion has to be performed with an incomplete set of data.

**Figure 6.**
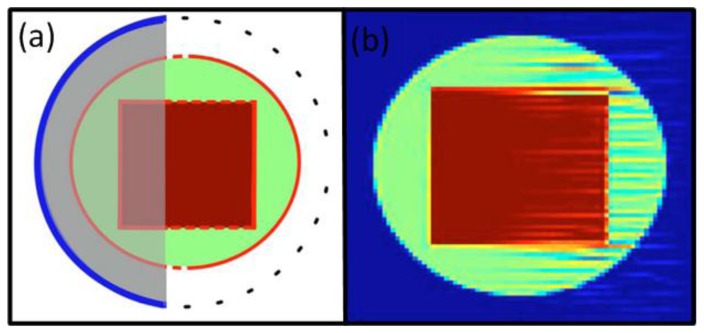
**(a)** Illustration of a limited view setup with detectors located on an open arc (solid blue line). Only the region covered by the detectors (shadowed area) can be reconstructed in a stable way. Sharp boundaries facing the detection surface (solid red lines) are visible whereas boundaries not facing the detection surface (dashed red lines) are invisible; (**b**) Reconstruction of (**a**) with a model-based inversion without regularization. Stripe artifacts arise due to the limited view geometry [116]. *Reprinted with permission from American Association of Physicists in Medicine*.

Xu *et al*. investigated the effects of limited view in an OAT setup with detectors on a truncated circle (Figure 6(a)) [115]. It has been found that regions enclosed by the detection surface can be recovered stably. Sharp boundaries of the object are thus visible in the reconstructions if they are facing the detection surface but turn invisible if they are perpendicular to the detection surface.

Buehler *et al*. showed that limited view reconstructions suffer from stripe artifacts (Figure 6(b)) [116]. The artifacts were reduced using a MB approach with a regularization term that suppresses ripples in the direction of the artifacts. Alternatively, total variation regularization terms can be applied in limited view scenarios to stabilize the inversion and reduce the artifacts [117]. Closed form reconstruction formulae can be also optimized for limited view scenarios. Paltauf *et al*. presented a BP approach that weights the signals with a smooth function before back-projecting [118]. The weighting function needs to consider whether complementary data from an opposite direction are available or these projections are missing. This approach may reduce artifacts resulting from the limited view while also preserving the simple BP implementation. Kunyansky presented an approach for non-closed detection surfaces based on single-layer potentials which, however, necessitates a non-trivial step of finding and calculating such potential [119]. Haltmeier *et al*. presented an efficient algorithm for a truncated planar detection geometry based on a nonuniform FFT that reduces the limited view artifacts while keeping computational efficiency of the fast Fourier methods [120].

### 3.6. Compressed Sensing Reconstruction Approaches

**Figure 7.**
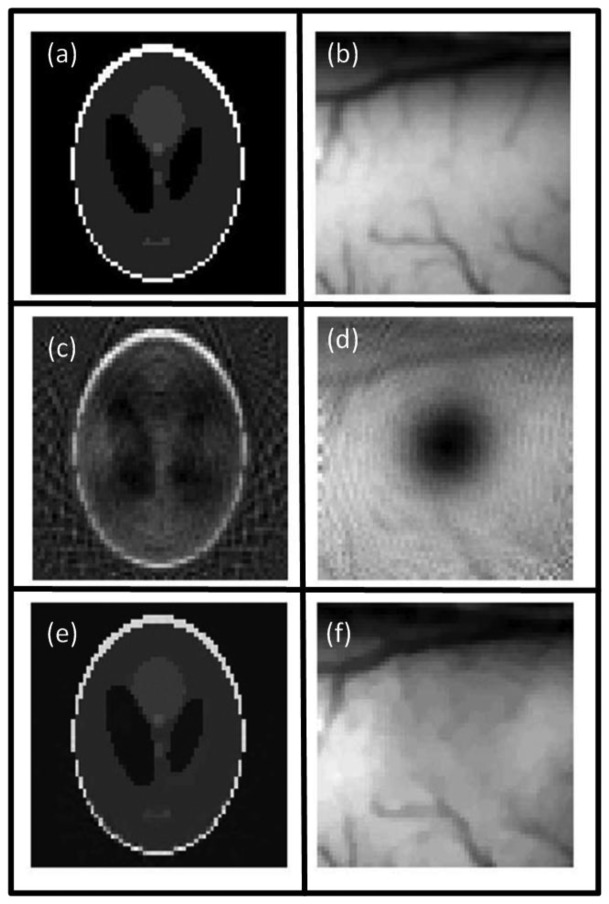
Reconstructions from highly undersampled data by Provost *et al*. [124]. The left and the right column show a Shepp-Logan phantom and a photograph of a cat brain, respectively. (**a**,**b**) Original images; (**c**,**d**) Reconstructions with pseudo-inverse of the model matrix; (**e**,**f**) Reconstructions using compressed sensing. *Reprinted with permission from IEEE*.

Recent reconstruction algorithms for OA have been also driven by the rapidly growing field of compressed sensing (CS) [121,122], which had been so far successfully applied to biomedical imaging in the field of MRI imaging [123]. Provost *et al*. were the first to suggest a CS scheme in OA imaging [124], followed by contributions from other groups [125,126]. The basic idea of CS is that images *p̃*_0_ can be represented by only a few large coefficients in a suitable basis instead of many small coefficients. CS exploits sparsity of the images based on a certain model *M̃* in that basis. Instead of minimizing the residual for an image in *L*_2_-norm (‖·‖_2_, Euclidian norm) only, CS also enforces the sparsity of the retrieved image by concurrently minimizing the *L*_1_-norm (‖·‖_1_, Taxicab norm) of the image. Thus, instead of Equation (9), one has to minimize:

(11) $${\tilde{p}}_{0}=\underset{{\tilde{p}}_{0}}{\text{argmin}}{\left\|\tilde{M}{\tilde{p}}_{0}-{\tilde{p}}_{d}\right\|}_{2}+\lambda \cdot {\left\|\Psi {\tilde{p}}_{0}\right\|}_{1}$$

where Ψ*p̃*_0_ is the representation of the image in the CS basis and *λ* is a scalar regularization parameter. Equation (11) can be solved by nonlinear minimization methods, which typically require longer calculation times as compared to just solving Equation (9). In comparison to standard image reconstruction methods, CS approaches have further shown reduction of artifacts in images when reconstructing from only a few tomographic projections (Figure 7). Thus, by reducing the number of acquired projections, these approaches hold great promise for dramatic reduction of image acquisition and reconstruction times as well as instrumentation costs.

## 4. Accounting for the Transport of Light

The last section has been entirely devoted to the acoustic part of the optoacoustic phenomena, *i.e.*, reconstruction of the initial pressure distribution and the deposited heat *H*(*r*) in the imaged object from the detected signals *p_d_*(*r_d_*, *t*). Nevertheless, understanding processes behind the optical counterpart governing propagation of the excitation light is equally important, mainly for two reasons. First, since photons are absorbed as they propagate in tissue, the depth at which a sufficient number of photons is left to excite a detectable signal limits the effective penetration depth of the imaging modality. Second, as stated earlier, the eventual goal of optoacoustic tomography is retrieving distribution of the optical absorption coefficient *μ_a_*(*r*) in the imaged object, which can be further related to distribution of the various chromophores and bio-markers in tissues. But the inversion process, described in the previous section, only retrieves maps of the deposited energy *H*(*r*), not *μ_a_*(*r*). Both distributions are related by the light fluence *Φ*(*r*, *μ_a_*, *μ_s_*, *g*) (see Equation (2)), which generally depends on the optical properties of the medium. In order to accurately quantify the values of the optical absorption coefficients and the corresponding concentrations of tissue chromophores, one needs to correct for the light fluence, which may vary considerably *versus* imaging depth. The reader is referred to a review article on that issue by Cox *et al.*, which has been published recently and covers the subject of image quantification in more detail [24].

### 4.1. Models of Light Transport

**Figure 8.**
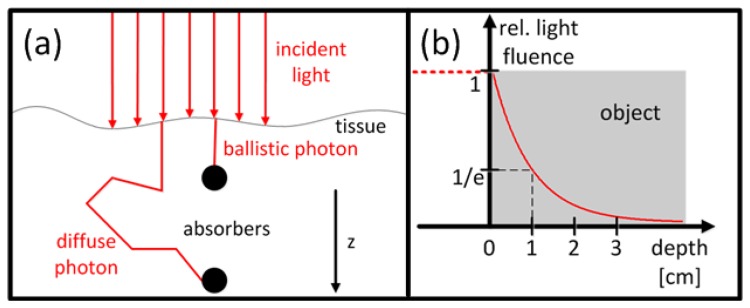
(**a**) Illustration of different light transport regimes. Light incident on tissue exhibits both scattering and absorption processes. Ballistic photons are absorbed close to the surface and are not scattered. Photons penetrating deep into the tissue are scattered multiple times before being absorbed and their propagation can be modeled by the light diffusion equation (LDE); (**b**) Depth profile of the light fluence with light incident on a highly scattering homogeneous object. For an effective absorption coefficient (*μ_eff_* = 1 *cm*^−1^) the light fluence drops exponentially to 1/*e* after 1 cm, limiting the effective penetration of light in tissue to few centimeters.

Several approaches exist to describe transport of light in diffusive tissues, which account for both absorption-which is mainly responsible for the contrast-and scattering processes. For visible and near-infrared light propagating in biological tissues, scattering can be typically considered as the dominant process. As depicted in Figure 8(a), one distinguishes between two main regimes of light transport: ballistic photons that have not been scattered and diffuse photons that have undergone multiple scattering events before being absorbed. At depths relevant for deep tissue OA tomography, typically between several millimeters to several centimeters, diffuse photons are dominant.

One frequently applied approach to model the transport of diffuse light is the Monte Carlo (MC) method, which simulates a random walk of a great number of photons through the scattering tissue [127–129]. The ensemble of all simulated paths results in the light fluence. Although MC calculations can be easily parallelized and optimized, they generally require an enormous calculation effort, which cannot be performed in real time.

An alternative description is given by the time-independent radiative transfer equation (RTE) derived from the Boltzmann equation (see Equation (9) in [24]). It models the light transport as integro-differential equation and holds for both ballistic and diffuse photons [83,130–132].

Because of the dominating scattering process, light transport in OA is most often modeled by a simpler approximation to the RTE, the so-called light diffusion equation (LDE) [133,134]:

(12) $$\mu_{a}(r)\cdot \Phi (\text{r})-\nabla \cdot (\text{D}(\text{r})\cdot \nabla )\Phi (\text{r})={\text{q}}_{0}(r)$$

where *q*_0_(*r*) is a source term and $D(r)={\left(3(\mu_{a}+\mu_{s}^{\prime })\right)}^{-1}$ is the diffusion coefficient (with the reduced scattering coefficient expressed via $\mu_{s}^{\prime }=(1-g)\mu_{s}$). For light incident upon a homogeneous scattering half-space, expression for the light fluence can be readily simplified as Φ(*z*) = Φ_0_ · exp (−*μ_eff_* · *z*) -well known as Beer's law-with an effective absorption coefficient $\mu_{\text{eff}}=\sqrt{3\mu_{a}(\mu_{a}+\mu_{s}^{\prime })}$. For instance, if *μ_eff_* = 1 cm^−1^, the light fluence will drop to 1/*e* of its maximal value after 1 cm (Figure 8 (b)). In this case, light penetration depth in tissue will be limited to a few centimeters.

If collimated light is incident on the object, the LDE is not accurate for depths close to the surface, where the light has not fully diffused. The *δ*-Eddington approximation is a higher order approximation to the RTE providing better results in the so-called mesoscopic region, typically up to a few millimeters in most tissues [135,136].

### 4.2. Model-Based Correction Schemes

Given a certain light transport model and *H*(*r*), Equation (2) needs to be solved for retrieving *μ_a_*. In general, this means solving a non-linear problem because *μ_a_* also non-linearly and implicitly depends on *H*(*r*) via the light fluence Φ(*r*, *μ_a_*, *μ_s_*, *g*). Various methods aim at obtaining both *μ_a_*(*r*) and *μ_s_*(*r*) simultaneously or only *μ_a_*(*r*) for a given *μ_s_*(*r*).

The solution is trivial for superficial imaging applications where only ballistic photons are of interest, e.g., as in case of optical resolution OA microscopy [137]. Alternatively, simple correction schemes, accounting for the drop of light fluence with depth, can be applied, e.g., analytical solutions to the LDE considering only the average optical properties of the medium [20].

A more complex light transport model can be also solved by an iterative fixed point calculation [135,136]. Here, the unknown *μ_a_*(*r*) is first written as an explicit function:

(13) $$\mu_{a}(r)=H(r)/\Phi (r,\mu_{a},\mu_{s},g)$$

A better approximation of the unknown *μ_a_*(*r*) is then obtained with each additional iteration and is subsequently used as an improved estimate for the right-hand term in the next iteration. Similarly, the problem can be treated in a linearized way by expanding *μ_a_* and Φ as a Taylor series (*μ_a_* = *μ_a_*_,0_ + *δμ_a_*, Φ = Φ_0_ + δΦ_0_). The problem can be linearly solved then for small perturbations *δμ_a_* and δΦ from their known solutions *μ_a,_*_0_ and Φ_0_. The fluence deviation *δ*Φ_0_ is assumed either as unchanged or as being linearly related to *δμ_a_* in the Born approximation. The solutions can be then obtained by a standard FE method [138,139].

Instead of separately treating the acoustic and the optical inverse problem, the solution can also be obtained in a hybrid manner. Laufer *et al*. presented a combined algorithm for modeling distributions of both the light (FE method) and the generated pressure field (k-space method) [140]. The method directly recovers the optical parameters quantitatively by iterative non-linear minimization of the difference between the computed and the measured pressure profiles.

Yet, optoacoustics is a high resolution imaging modality, which makes accurate modeling of the light transport and quantification of OA images challenging. If the various models and equations used in the reconstruction process are not too far away from the experimental reality, the estimate for *μ_a_*(*r*) is expected to converge to a stable solution and obtain a good approximate after only a few iterations. In some other cases, solution for the absorption coefficient was shown to be divergent [138] or otherwise the solution was found to be non-unique and ill-posed when trying to recover *μ_a_* and *μ_s_* simultaneously [141].

### 4.3. Other Approaches

The importance of knowing the light fluence in real experiments and the challenges with modeling light propagation in optically heterogeneous tissues have led to some alternative approaches attempting to correct for light attenuation.

One method to increase the amount of available information regarding optical properties of the imaged tissues is to use multi-illumination patterns [139,141,142]. The object is illuminated from different angles with light of the same wavelength. Bal *et al*. presented an approach that can—under some assumptions—recover the optical properties from a multi-illumination pattern in a non-iterative way [35]. Alternatively to multi-illumination patterns, the inversion process can also be done simultaneously for multi-wavelength illumination with knowledge on the spectral dependence of the optical parameters (see next section).

**Figure 9.**
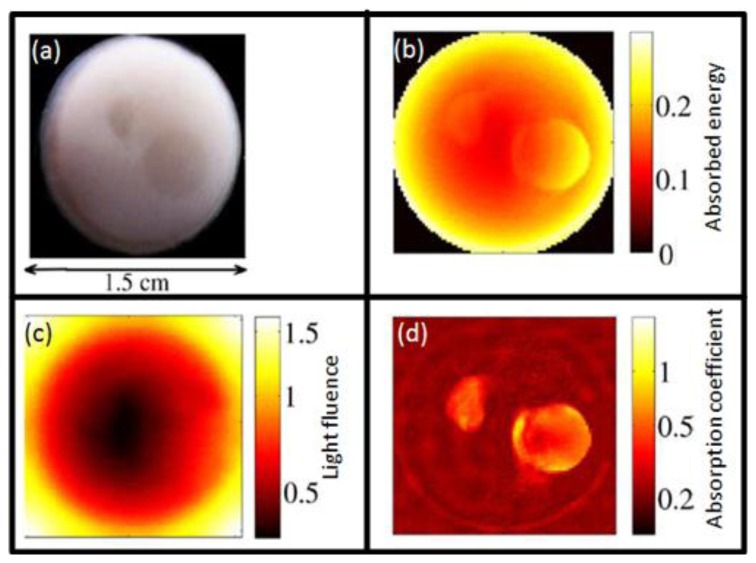
Sparse decomposition of an OA reconstruction presented by Rosenthal *et al*. to determine both the light fluence and the absorption coefficient [143]. (**a**) Schematic description of the phantom used; (**b**) OA reconstruction of the deposited heat in the phantom; (**c**) Recovered light fluence represented sparsely in a Fourier basis; (**d**) Recovered absorption coefficient represented sparsely by Haar wavelets.

Rosenthal *et al*. presented a blind separation approach which does not consider any particular light transport model [143]. Instead, it is based on a fairly realistic assumption that the light fluence Φ(r) is a slowly varying function of space whereas the absorption coefficient *μ_a_*(*r*) has mainly high spatial frequency components. The idea is to sparsely represent a logarithmic representation of the reconstructed optoacoustic image log *H*(*r*) in a set of two bases, a wavelet basis Λ*_n_*(*r*) and a Fourier basis Ψ*_m_*(*r*), *i.e.*,

(14) $$\log H(r)=\log\mu_{a}\text{(}r)+\log\Phi (r)=\sum_{n=1}^{N}\alpha_{n}\cdot \Lambda_{n}(r)+\sum_{m=1}^{M}\beta_{m}\cdot \Psi_{m}(r)$$

where the coefficients *α_n_* are attributed to the absorption coefficient *μ_a_*(*r*) and *β_m_* to the light fluence Φ(r). In this way, both the absorption and the light fluence can be reconstructed from the OA image blindly (Figure 9), without modeling the light propagation in tissues.

Cox *et al*. suggested a method that directly ‘measures’ the local light fluence [144]. Its key component is a chromophore whose absorption has a highly non-linear dependence on the light fluence at a certain threshold intensity. With increase of the illumination intensity, the chromophores are ‘switched’ on (or off) at positions where the local light fluence crosses that threshold. The applicability of this approach however requires the availability of a contrast agent with such highly non-linear absorbance.

Another approach is to experimentally determine the light fluence using a hybrid imaging approach that also accommodates diffuse optical tomography (DOT) [145–147]. Because of their purely optical nature, DOT images are heavily affected by light diffusion thus cannot provide the same spatial resolution as OA. But they can be instead used to improve quantification of OA images. Maslov *et al.* implanted an absorber with known optical parameter in tissue at depth to investigate the effects of light fluence [148]. Although being an interesting experimental exercise, this invasive method seems impractical for *in-vivo* imaging applications.

## 5. Multispectral Processing in Optoacoustic Imaging

**Figure 10.**
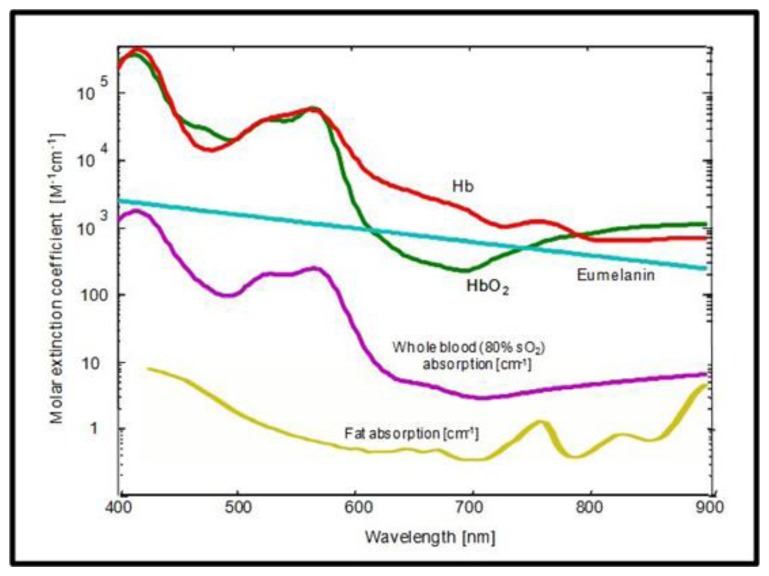
Spectra of dominant intrinsic absorbers in biological tissue in the visible and near-infrared range. Chromophores can be distinguished by their different spectral dependence of the absorption coefficient on the wavelength (data taken from [20,153]).

The contrast in OA stems from differential light absorption and can be of either intrinsic or extrinsic nature. Figure 10 shows the absorption spectra of the most dominant intrinsic absorbers in tissue in the visible and near-infrared range.

For many applications, the blood-related absorption is of particular interest, providing valuable physiological or functional information. The main challenge arises from the fact that, at any given wavelength, more than one chromophore contributes to the total absorption coefficient *μ_a_*(*r*). For instance, at 800 nm, the oxygenated (HbO_2_) and the deoxygenated (Hb) forms of hemoglobin contribute equally to absorption and thus are indistinguishable using single wavelength measurements. Therefore, it is the concentrations of the different chromophores *c_i_*(*r*), not the total absorption *μ_a_*(*r*), which is mainly of interest from the biological point of view. This relation may be expressed via linear superposition of the different chromophores:

(15) $$\begin{array}{cc} \mu_{i}(r,\lambda )=\sum_{i}\varepsilon_{i}(\lambda )\cdot c_{i}(r) & \left[+\mu_{\text{back}}(r,\lambda )\right] \end{array}$$

where *ϵ_i_*(*λ*) are their wavelength-dependent molar extinction coefficients and *μ_back_*(*r*, *λ*) is the residual (background) absorption, which might also include noise. To differentiate between contributions of different chromophores, their distinct spectral dependence on wavelength can be assessed [11,149]. This multi-wavelength approach is known as multispectral optoacoustic tomography (MSOT) or spectroscopic imaging [148,150–152]. The process of recovering *c_i_*(*r*) from multispectral measurements is known as unmixing. It can be combined with calculation of the light fluence or treated as a separate image processing step.

Due to versatility and wide availability of optical molecular agents, sensitive and accurate spectral processing to recover concentration of extrinsically-administered agents may enable longitudinal molecular imaging studies. This can be done by resolving accumulation of agents with specific spectral signatures, such as targeted and activatable fluorescent molecular agents, nanoparticles or genetic markers.

### 5.1. Unmixing with Known Spectra

If spectra of the different components (chromophores) are known, their concentrations can be directly calculated from the OA absorption images. In some special cases this step is rather trivial, e.g., if the certain chromophore of interest is entirely dominant over other absorbers at a given wavelength or if one is only interested in the total blood concentration measured at the isobestic point, for which Hb and HbO_2_ are equally absorbing.

The simplest multispectral OA imaging approach was presented by Kruger *et al*. and consisted of subtracting the images obtained at two different wavelengths [13]. It was used to recover distribution of an optical agent, having sharp variations in its absorption spectrum, over wavelength-independent absorbing background. A similar approach proved efficient for detection of dynamic contrast based on a pump-probe excitation [154,155]. Here, a phosphorescent chromophore was pumped to an excited state with a laser pulse at the pump wavelength. The transient absorption of the excited chromophore was then probed with a slightly delayed second laser pulse with a different wavelength. The dynamic contrast can be extracted by subtracting the pump-only absorption from the combined pump-probe absorption. Also, it is well known that blood oxygenation level *saO*_2_ is independent of the total concentration of Hb and HbO_2_ but only depends on their ratio, *i.e.*,

(16) $$saO_{2}=\frac{c_{HbO_{2}}}{c_{HbO_{2}}+c_{Hb}}$$

Consequently, the oxygenation level can in principle be obtained from the ratio of OA images acquired at two different wavelengths.

Despite its relative simplicity, the image subtraction method cannot provide quantitative measurements of distribution of several chromophores. The approach can be thus generalized to an arbitrary number of chromophores with unknown concentrations and known spectra [44,45]. In this way, for a given set of wavelengths, the concentrations are linearly fitted to Equation (15) on a per-voxel basis using a linear regression method. This can be efficiently realized with a matrix relation:

(17) $$c_{i}=\sum_{\lambda }S_{\text{i}\;\lambda }^{†}\cdot \mu_{\lambda }$$

where *c_i_* and *μ_λ_* are respectively the concentrations of the components and the absorption coefficient in a certain reconstructed image voxel. $S_{i\lambda }^{†}$ is the Moore-Penrose pseudo-inverse of the spectra matrix *S* derived from the components' molar extinction coefficients. In general, the number of measured wavelength should be greater than the number of chromophores.

### 5.2. Blind Unmixing Methods

In many cases, the absorption spectra of all the absorbing components present in the imaged object may not be exactly known. As a result, the unmixing method needs to simultaneously retrieve both the chromophore concentrations and their spectra. In contrary to the previously presented methods, the so-called blind unmixing methods do not operate on a per-voxel basis but exploit the statistical properties of a set of images without requiring prior knowledge of the spectra.

**Figure 11.**
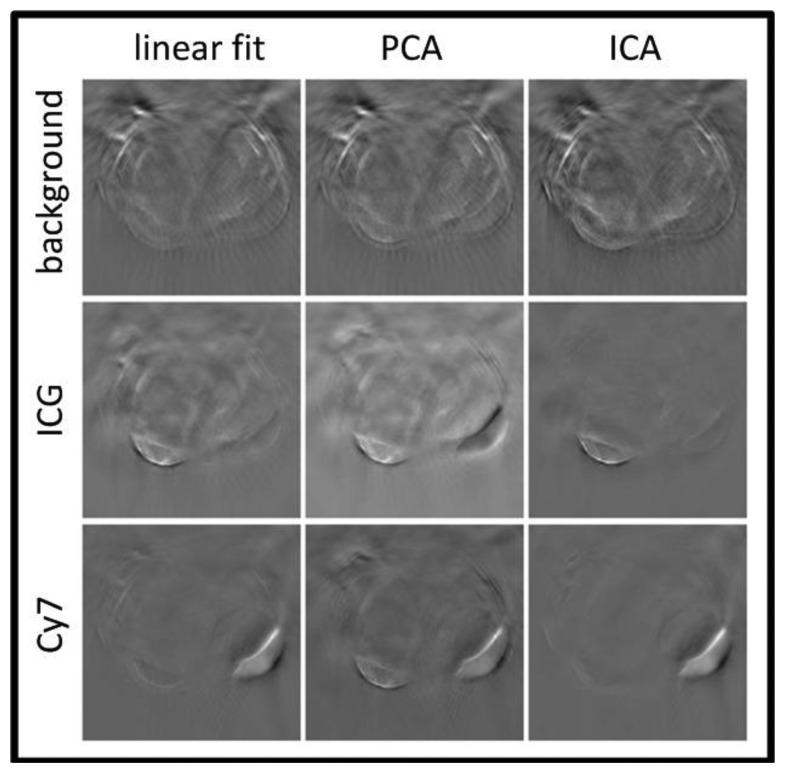
Comparison of the unmixing performance for different algorithms by Glatz *et al*. [156]. They implanted two insertions containing ICG and Cy7 in the neck area of an euthanized mouse and imaged the region multispectrally. The rows (top to bottom) show the components corresponding to background, ICG and Cy7, respectively. The columns show (left to right) the performance of linear fitting from known spectra and of blind methods with a PCA and an ICA algorithm, respectively. *Reprinted with permission from Optical Society of America*.

Glatz *et al*. investigated the performance of blind spectral unmixing on OA images using two multivariate methods [156]. Principal component analysis (PCA) is based on the assumption that the different chromophore distributions are statistically uncorrected [157] and yields an orthonormal transformation into a new base, in which the largest data variance is projected onto the first principal component, the largest remaining variance onto the second one, and so on. In this way, the spectrally correlated measurement data are unmixed by being transformed to the uncorrected components. Similarly, independent component analysis (ICA) is based on a more general assumption that the source components are statistically independent [158]. Consequently, the spectrally mixed measurement data are transformed to statistically independent source components. Both methods are able to recover the spectra of the different components via the transformation matrix and their concentrations via the transformed images. Figure 11 summarizes the results obtained with the linear fitting, PCA and ICA algorithms.

## 6. Selected Biomedical Applications

In this section, we shortly highlight several representative examples of the emerging optoacoustic imaging applications while a more comprehensive review on biomedical applications of optoacoustics can be gained from other recently published review articles [15,21,22,152].

**Figure 12.**
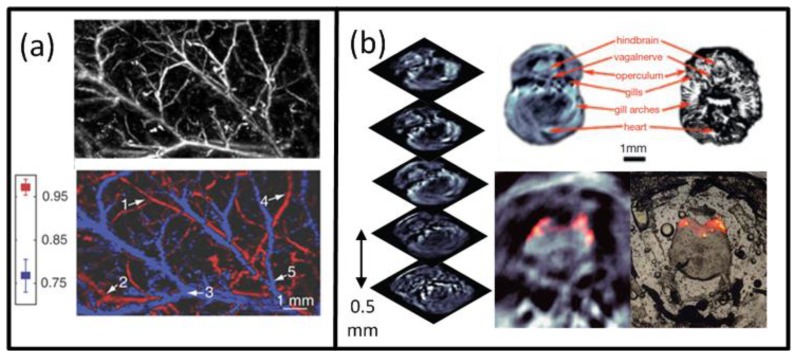
(**a**) Functional OA microscopy by Zhang *et al*. [44]. Structural *in vivo* image of rat vasculature taken at 584 nm (top). Functional vessel-by-vessel *saO*_2_ mapping based on a linear fitting of four different wavelengths (bottom). *Reprinted with permission from Nature Publishing Group*; (**b**) *In vivo* detection of fluorescent proteins by Razansky *et al.* [45]. OA images of the hindbrain area of a mCherry expressing zebra fish for five different slices taken at 585 nm (left). Selected slice and its corresponding histological section (top right). MSOT image of the brain with mCherry expression shown in color and its corresponding epi-fluorescent histology (bottom right). *Reprinted with permission from Nature Publishing Group*.

Naturally, the best intrinsic tissue contrast arises from highly absorbing hemoglobin, thus blood is clearly visible in the images. Functional OA microscopy is thus geared towards investigation of vascular structures, providing an excellent intrinsic contrast and a high spatial resolution in the order of some tens of μm and penetration of several millimeters into scattering tissues. In order to obtain high resolution 3-D images, a single spherically-focused high frequency detection element is mechanically raster-scanned along a plane [30,44,137,159]. Significantly higher spatial resolution is further achieved by using a tightly focused optical illumination spot, resulting in the so-called optical resolution microscopy. Despite providing much better spatial resolution in the sub-micron range, similarly to all the other optical microscopy techniques, the latter method suffers from limited penetration depth of only several hundreds of microns due to intense photon scattering in biological tissues. An example of a typical acoustic resolution *in vivo* functional optoacoustic microscopy system [44] is shown in Figure 3(a). A tunable laser generated pulses of 6-ns duration which passed through a fiber and formed a ring-shaped illumination pattern to suppress OA signals from the surface. A coaxially aligned focused 50-MHz central frequency detector acquired time-resolved one-dimensional signals. The transducer was scanned in 50 μm steps in the x-y plane to form a 3-D image without an inversion algorithm needed. With this setup, subcutaneous vasculature of a Sprague-Dawley rat was demonstrated. Figure 12(a) shows the structural image obtained at the isobestic wavelength of 584 nm in grayscale and the functional *saO*_2_ image obtained by a linear fitting to four different wavelengths in color. The *saO*_2_ levels obtained were 0.97 and 0.77 for arterial and venous blood, respectively. Changes in *saO*_2_ for hypoxic and hyperoxic conditions of the animal were successfully tracked, offering interesting prospects for non-invasive functional brain imaging.

Preclinical whole-body imaging of small animals with multispectral optoacoustic tomography (MSOT) systems is yet another key application of OA imaging. These systems can provide tomographic images with a resolution in the order of hundred μm for depths between several millimeters to several centimeters. Possible applications include monitoring of tumor hypoxia, drug response or molecular targets in biological model organisms [14,20,45,160]. The ability of MSOT to visualize deep-seated fluorescent proteins with high resolution has also been demonstrated [45]. The setup utilized a selective-plane light illumination and a confocal ultrasound detection pattern (Figure 3(b)). An OPO laser provided 8-ns laser pulses of tunable wavelength and the signals were captured by rotating a 3.5-MHz central frequency transducer around the object in 3° steps for multiple slices. The images for multiple wavelengths were reconstructed with the UBP algorithm and unmixed with a linear fitting on a per-pixel basis. In this way, a transgenic three-month old zebra fish expressing mCherry fluorescent proteins in the vertebral column was visualized *in vivo*. Figure 12(b) shows the structural OA image of the hindbrain at 585 nm and its corresponding histological slice (top row). The multispectral OA imaging has accurately attained the mCherry expression, which has also well corresponded with the findings from epi-fluorescent histology (bottom row).

**Figure 13.**
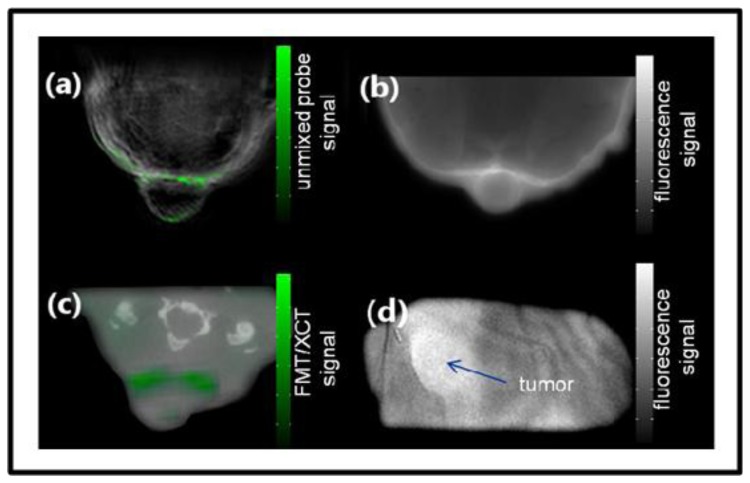
Comparison between optical imaging and MSOT for imaging of a targeted apoptotic marker (PSS-794) in 4T1 tumor-bearing mice by Buehler *et al*. [161]. (**a**) High resolution MSOT image shows the superposition of a single-wavelength (anatomical) optoacoustic image (in gray scale) and the unmixed component corresponding to the PSS-794 signal (in color); (**b**) Corresponding epi-fluorescent image; (**c**) Reconstruction from a hybrid FMT/XCT modality; (**d**) Planar trans-illumination image. *Reprinted with permission*.

Buehler *et al*. presented the capabilities of MSOT for targeted molecular imaging in real time [161]. In the *in vivo* mouse experiments, a ring-shaped illumination was provided by a tunable OPO (680–950 nm, 10-ns, 10 Hz) and delivered through a fiber bundle. A 64 element transducer array (5-MHz central frequency) covering 172° solid angle detected the signals in parallel without multiplexing. The CD-1 nude mice containing 4T1 tumor allografts were wrapped in a transparent membrane to prevent direct contact of the mouse with water. A near-infrared apoptosis targeting probe (PSS-794, 50-100 nanomoles [162]) was injected in three mice at different time points. The images were reconstructed using a model-based algorithm and unmixed with an ICA spectral unmixing algorithm. Figure 13(a) shows the MSOT image containing both high resolution anatomical and functional information for a tumor-bearing mouse with PSS-794 probe. From the MSOT images (Figure 13(a)), it was clearly determined that the PSS-794 probe mainly accumulated in the blood vessels surrounding the tumors and did not infiltrate into the tumor mass. On the other hand, the use of three-dimensional optical tomography (FMT) has not attained the sufficient spatial resolution that would determine the precise location of the probe (Figure 13(c)), mainly due to its poor spatial resolution resulting from photon scattering.

**Figure 14.**
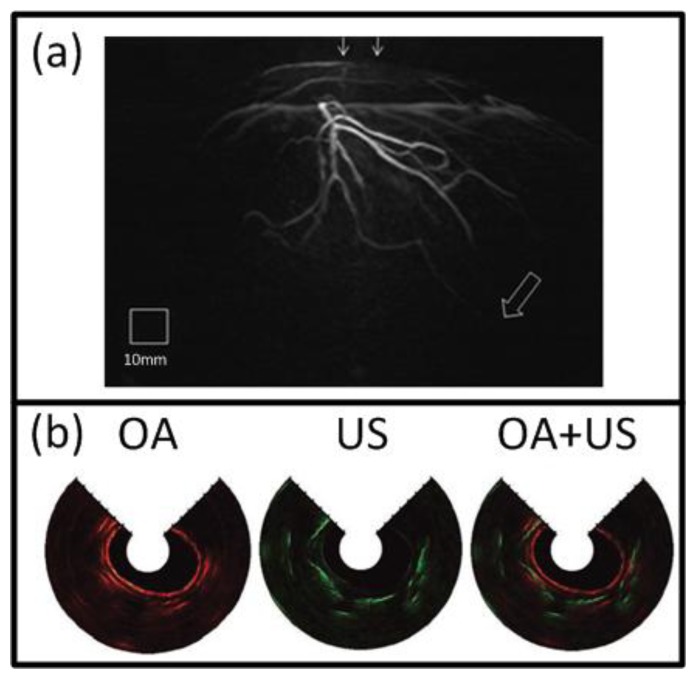
(**a**) OA angiography of the breast by Kruger *et al*. without contrast enhancement [49]. Maximum intensity projection of a 57 year old volunteer's breast showing the vasculature from superficial areas (solid arrows) up to considerable depth (hollow arrow). *Reprinted with permission from American Association of Physicists in Medicine*; (**b**) Simultaneous, dual-wavelength OA and US endoscopy by Yang *et al*. [47]. Ultrasonic, optoacoustic and combined cross-sectional *in vivo* images of a rabbit esophagus near the lungs taken at 584 nm. *Reprinted with permission from Nature Publishing Group*.

At present, the great potential of optoacoustic imaging that was showcased in preclinical research has encouraged translation of this technology into clinical practice. The clinical promise of OA is greatly supported by its non-ionizing radiation, rich contrast, real-time operation, and relatively low cost. It is likely that OA will increasingly enter the clinical imaging segments in areas such as skin, breast, vasculature, and ocular imaging, visualization of tumor and inflammation-related pathology *etc*. [17,58,163,164]. Indeed, early detection of breast cancer will capture a significant spot in the future developments of OA imaging applications [25,49,50]. One example of such clinical system for OA breast angiography without the need for a contrast agent was recently presented [49]. The system employed 128 detection elements (5-MHz central frequency) which were placed on a 100 mm radius hemisphere in a spiral pattern and acquired the signals in parallel (Figure 3(f)). The hemisphere was rotated for higher projection sampling and one complete scan took between 6 to 24 s. The volunteer's breast was immersed into a water-filled bowl from the top and immobilized by an optically and acoustically transparent cup. 3-D images were reconstructed from a filtered BP algorithm with prior frequency filtering and transducers' impulse response deconvolution. The system was used to visualize the vasculature of a 57 year-old volunteer without a contrast agent and vessels from superficial regions up to a considerable depth of several centimeters (Figure 14(a)).

Naturally, clinical application of OA imaging is limited to regions that can be efficiently illuminated with the excitation light. When the imaged region of interest is not reachable from the outside, certain areas of the human body can also be imaged with minimally invasive catheter- or endoscopy-based techniques. To this end, such systems have been realized for other imaging modalities, such as intra-vascular ultrasound (IVUS) [165] or fluorescent imaging (NIRF) [166]. In the recent years, similar approaches have been also attempted with OA for intravascular, esophageal or colonoscopic imaging [47,151,167–169]. One example is shown in Figure 3(d), where a system for simultaneous functional OA and US endoscopy *in vivo* is presented [47]. The endoscope was able to acquire co-registered pulse-echo US and OA images. A-scans were obtained from an ultrasonic pulse-echo system while two OA pulses, having different wavelengths for spectroscopic imaging, were delivered through a fiber and detected by the focused transducer. 3-D images could be obtained by constant rotation of a scanning mirror and a mechanical pullback of the catheter. Using this system, rabbit esophagus was imaged *in vivo*, as shown in Figure 14(b).

## 7. Limitations and Future Challenges of Optoacoustic Imaging

Indeed, optoacoustic imaging offers multiple advantages over other imaging modalities. Most favorable is the combination of US-diffraction limited spatial resolution and excellent optical absorption contrast without any ionizing radiation involved. Clearly, much like all other imaging modalities, optoacoustics is limited in certain aspects. As presented in the current review, due to the (non-ideal) highly heterogeneous and wavelength-dependent nature of biological tissues, images reconstructed from experimental data usually contain artifacts, such as negative values in the images which otherwise have no physical meaning. In addition, high noise levels, limited bandwidth of the detection system, inaccurate assumptions on the speed of sound, acoustic attenuation, as well as limited view in detection, can all alter the signals in a way that the reconstructed image accuracy is highly compromised. While some simple algorithms, such as Hilbert transform for envelope detection or thresholding of negative values, can be used to keep image values in the positive region, these non-linear operations in fact only conceal the underlying problem while not assisting with achieving quantitative results. Instead, reliable detection and modeling of signal propagation in tissues is required.

Imaging at depth brings further challenges and limitations. First, imaging depth is compromised by attenuation of the light fluence in optically opaque tissues. Some of it can be compensated by increasing the amount of the deposited laser energies. However, for *in vivo* applications in the near-infrared, illumination on the skin surface is limited by the maximum permissible exposure (MPE, [170]) to about 20 mJ/cm^2^. As a result, imaging depth is usually restricted to regions where the light fluence is sufficiently high to generate detectable pressure variations, typically up to a few centimeters in most soft tissues. In addition, even though spatial resolution of optoacoustic imaging is not affected by light scattering, resolution may still deteriorate with increasing imaging depth due to its dependence on the frequency content of the detected ultrasonic responses. This is because of the dispersive nature of ultrasound waves with high frequency components being rapidly attenuated as they propagate through tissues [171].

In multispectral imaging applications, one also faces the problem of so-called ‘spectral coloring’. Due to the non-local and non-linear dependence of light transport on the object's optical properties, spectra of various tissue chromophores and agents, extracted by means of optoacoustics, might be corrupted. For improving quantitative determination of chromophore concentrations using their known spectra, an accurate correction for the light distribution needs to be employed, which, as discussed before, constitutes a challenging problem and an open area of research. Another important aspect influenced by depth is the limits of the imaging system with respect to detection of minimal concentrations of certain intrinsic tissue chromophores and extrinsically-administered contrast agents. To this end, several publications have reported sensitivity in the femtomole to picomole range, depending on the molecular weight of the probe employed [20,28], while a recent study has also systematically addressed the question of how the detection limits are affected by imaging depth [172]. In reality, sensitivity limits are affected by multiple additional factors, such as the total volume, spectrum and absorption coefficient of the imaged chromophore, the noise equivalent pressure (NEP) of the detectors, level and spectral dependence of background tissue absorption.

Instrumentation-related limitations are an additional factor in translating optoacoustic imaging methods into routine application. One of the important advantages, which helps reducing motion-related image artifacts but may also enable powerful applications in *in-vivo* tracking of dynamic processes, is the possibility for reliable measurements and image rendering in real-time. This requires introducing high repetition pulsed laser technology and fast wavelength tuning capabilities, especially for multi-spectral imaging applications involving real-time visualization of distributions of spectrally distinct contrast agents. On the other hand, the detection technology should become increasingly sensitive but also parallelized to enable real-time image acquisition. Often a trade-off between acquisition speed and quality on the one hand and costs of instrumentation on the other hand has to be found. In this respect, optoacoustic imaging has greatly benefited from the advances in both laser and detection technology over the last years and significant performance enhancements are expected as the technological frontiers advance. Providing information from hybrid US imaging will be of added value for future clinical systems. Besides fast data acquisition, fast image rendering is also of great importance to readily provide findings for efficient real-time guidance and optimization of diagnostic or therapeutic procedures. Recently, extensive research is devoted to the subject of real-time visualization, benefiting both from algorithmic developments and advances in parallel computing technology.

Quantitative rendering of chromophore concentration is perhaps among the most important but also among the most challenging tasks of the optoacoustic methods. Hemoglobin in its oxygenated and deoxygenated form is omnipresent in tissue and is the most dominant intrinsic absorber in both the visible and most of the near-infrared range of the optical spectrum. Thus, quantitative determination of blood parameters is of great interest as it is related to a variety of physiological parameters. Prominent examples are monitoring angiogenesis or hypoxic states in tumors [173], functional imaging in the brain [44] or responses to environmental changes [174]. In most cases, the oxygen saturation *saO*_2_ (Equation (16)) is of main interest, whose quantitative determination is non-trivial, especially in the presence of strong wavelength dependence of the excitation light fluence in tissues. First attempts to reliably determine *saO*_2_ were conducted *in vitro* [175,176] whereas accuracy of ±2.5% *saO*_2_ and a minimum detectable change of ±1% *saO*_2_ were reported [176]. Furthermore, oxygenation saturation of single blood vessels, embedded deep in tissue mimicking phantom, was determined using numerical forward light transport model, reporting an accuracy of ±7% *saO*_2_ [150]. Most *in vivo* results were so far obtained from multispectral optical resolution optoacoustic microscopy where light transport issues can be effectively omitted. Zhang *et al*. reported 4% *saO*_2_ systematic error for their *ex vivo* study with bovine blood, while accuracy for *in vivo* determination of arterial and venous blood under hypoxic, normal and hyperoxic conditions was not reported [177]. Applications of efficient and practical *saO*_2_-determination schemes for experimental data in 3-D in an accurate, robust and computationally feasible manner presents therefore one of the key challenges for the current algorithmic developments. Finally, when imaging other chromophores or extrinsically-administered contrast agents, the blood background needs to be accounted for to enable quantified extraction of biomarker concentrations.

## 8. Conclusions

Owing to its hybrid nature, *i.e.*, optical excitation and ultrasonic detection, optoacoustics benefits from both rich and versatile optical contrast and high (diffraction-limited) spatial resolution, associated with relatively low scattering of ultrasonic waves. Optoacoustic biosensing and imaging provides an excellent platform for multi-scale investigations using the same contrast, from microscopic observations at the single capillary and cell level to whole body imaging of small animals and deep tissue imaging of humans. Much like other optical imaging modalities, optoacoustics is safe for both small animals and clinical use as it utilizes non-ionizing radiation at the visible and near-infrared wavelengths.

In the last decade, OA imaging is considered to be the fastest growing biomedical imaging modality with multiple already implemented and envisioned applications in biomedical research and clinical practice, from diagnostic applications in cancer research and brain imaging to drug development and treatment monitoring. Clearly, progress of the image reconstruction and quantification methods has been central to those developments and has significantly contributed to creation of new exciting applications of the OA imaging technologies. As discussed in this review, multiple frontiers are still open in the algorithmic areas, where many challenges related to removal of image artifacts, image quantification, reconstruction strategies in the presence of acoustically mismatched areas, real-time operation, and multi-spectral data processing need to be further addressed.

## Figures and Tables

**Table 1.** Comparison of different sound detection technologies used in optoacoustic imaging.

	Piezoelectric	Optical	Capacitive
Bandwidth			
Sensitivity			
Miniaturization			
Cost efficiency			
Parallelization			
Refs	[50,51]	[46,53,54]	[55-57]

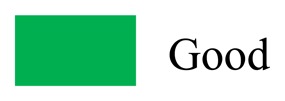	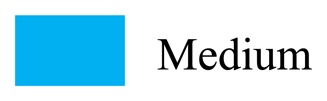	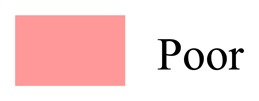	

## References

[1] Tainter C.S., Bell A.G. (1880). Selenium and the photophone. Nature.

[2] Bell A.G. (1881). Production of sound by radiant energy. Manuf. Build..

[3] Bell A.G. (1881). The spectrophone. Bull. Phil. Soc..

[4] Tyndal J. (1880). Action of an intermittent beam of radiant heat upon gaseous matter. Proc. R. Soc. Lond..

[5] Röntgen W.C. (1881). On tones produced by the intermittent irradiation of a gas. Philos. Mag..

[6] Groth M. (1987). Photophones revisted. Aust. Melb..

[7] Veingerov M.L. (1938). New method of gas analysis based on Tyndall-Röntgen optic-acoustic effect. Dokl. Akad. Nauk. USSR..

[8] Delany M.E. (1959). The optic-acoustic effect in gases. Sci. Prog..

[9] Bobylev B.A., Kravchenko A.F. (1969). Photoacoustic spectra of high-resistivity gallium arsenide. Fizika i Tekhnika Poluprovodnikov..

[10] Rosencwaig A. (1973). Photoacoustic spectroscopy of solids. Opt. Commun..

[11] Rosencwaig A. (1973). Photoacoustic spectroscopy of biological materials. Science.

[12] Oraevsky A.A., Jacques S.L., Esenaliev R.O., Tittel F.K. (1994). Laser based optoacoustic imaging in biological tissues. Proc. SPIE.

[13] Kruger R.A., Kiser W.L., Reinecke D.R., Kruger G.A., Miller K.D. (2003). Thermoacoustic molecular imaging of small animals. Mol. Imaging..

[14] Wang X.D., Pang Y.J., Ku G., Xie X.Y., Stoica G., Wang L.H.V. (2003). Noninvasive laser-induced photoacoustic tomography for structural and functional *in vivo* imaging of the brain. Nat. Biotechnol..

[15] Ntziachristos V., Razansky D. (2010). Molecular imaging by means of multispectral optoacoustic tomography (MSOT). Chem. Rev..

[16] Ntziachristos V., Ripoll J., Wang L.H.V., Weissleder R. (2005). Looking and listening to light: The evolution of whole-body photonic imaging. Nat. Biotechnol..

[17] Zhang E.Z., Laufer J.G., Pedley R.B., Beard P.C. (2009). *In vivo* high-resolution 3D photoacoustic imaging of superficial vascular anatomy. Phys. Med. Biol..

[18] Song L., Maslov K., Wang L.V. (2010). Section-illumination photoacoustic microscopy for dynamic 3D imaging of microcirculation. in vivo. Opt. Lett..

[19] Razansky D., Vinegoni C., Ntziachristos V. (2009). Imaging of mesoscopic-scale organisms using selective-plane optoacoustic tomography. Phys. Med. Biol..

[20] Razansky D., Vinegoni C., Ntziachristos V. (2007). Multispectral photoacoustic imaging of fluorochromes in small animals. Opt. Lett..

[21] Beard P. (2011). Biomedical photoacoustic imaging. Interface Focus.

[22] Wang L.H.V., Hu S. (2012). Photoacoustic tomography: *In vivo* imaging from organelles to organs. Science.

[23] Kuchment P., Kunyansky L. (2008). A survey in mathematics for industry mathematics of thermoacoustic tomography. Eur. J. Appl. Math..

[24] Cox B., Laufer J.G., Arridge S.R., Beard P.C. (2012). Quantitative spectroscopic photoacoustic imaging: A review. J. Biomed. Opt..

[25] Kruger R.A., Miller K.D., Reynolds H.E., Kiser W.L., Reinecke D.R., Kruger G.A. (2000). Breast cancer *in vivo*: Contrast enhancement with thermoacoustic CT at 434 MHz-Feasibility study. Radiology.

[26] Razansky D., Kellnberger S., Ntziachristos V. (2010). Near-field radiofrequency thermoacoustic tomography with impulse excitation. Med. Phys..

[27] Amiot C.L., Xu S.P., Liang S., Pan L.Y., Zhao J.X.J. (2008). Near-infrared fluorescent materials for sensing of biological targets. Sensors.

[28] Ku G., Wang L.H.V. (2005). Deeply penetrating photoacoustic tomography in biological tissues enhanced with an optical contrast agent. Opt. Lett..

[29] Tong L., Wei Q.S., Wei A., Cheng J.X. (2009). Gold nanorods as contrast agents for biological imaging: Optical properties, surface conjugation and photothermal effects. Photochem. Photobiol..

[30] De La Z.A., Zavaleta C., Keren S., Vaithilingam S., Bodapati S., Liu Z., Levi J., Smith B.R., Ma T.J., Oralkan O. (2008). Carbon nanotubes as photoacoustic molecular imaging agents in living mice. Nat. Nanotechnol..

[31] Zimmer M. (2002). Green fluorescent protein (GFP): Applications, structure, and related photophysical behavior. Chem. Rev..

[32] Maslov K., Wang L.V. (2008). Photoacoustic imaging of biological tissue with intensity-modulated continuous-wave laser. J. Biomed. Opt..

[33] Telenkov S.A., Mandelis A. (2008). Fourier-domain methodology for depth-selective photothermoacoustic imaging of tissue chromophores. Eur. Phys. J. Spec. Top..

[34] Kellnberger S., Deliolanis N.C., Queiros D., Sergiadis G., Ntziachristos V. (2012). *In vivo* frequency domain optoacoustic tomography. Opt. Lett..

[35] Bal G., Ren K. (2011). Multi-source quantitative photoacoustic tomography in a diffusive regime. Inverse Probl..

[36] Bal G., Ren K. (2012). On multi-spectral quantitative photoacoustic tomography in diffusive regime. Inverse Probl..

[37] Larina I.V., Larin K.V., Esenaliev R.O. (2005). Real-time optoacoustic monitoring of temperature in tissues. J. Phys. D Appl. Phys..

[38] Pramanik M., Wang L.V. (2009). Thermoacoustic and photoacoustic sensing of temperature. J. Biomed. Opt..

[39] Kruger R.A., Liu P., Fang Y.R., Appledorn C.R. (1995). Photoacoustic ultrasound (PAUS)— reconstruction tomography. Med. Phys..

[40] Ma R., Söntges S., Shoham S., Ntziachristos V., Razansky D. (2012). Fast scanning coaxial optoacoustic microscopy. Biomed. Opt. Express..

[41] Zemp R.J., Song L.A., Bitton R., Shung K.K., Wang L.H.V. (2008). Realtime photoacoustic microscopy *in vivo* with a 30-MHz ultrasound array transducer. Opt. Express..

[42] Allen T.J., Beard P.C. (2006). Pulsed near-infrared laser diode excitation system for biomedical photoacoustic imaging. Opt. Lett..

[43] Wang Y., Maslov K., Zhang Y., Hu S., Yang L.M., Xia Y.N., Liu J.A., Wang L.H.V. (2011). Fiber-laser-based photoacoustic microscopy and melanoma cell detection. J. Biomed. Opt..

[44] Zhang H.F., Maslov K., Stoica G., Wang L.H.V. (2006). Functional photoacoustic microscopy for high-resolution and noninvasive *in vivo* imaging. Nat. Biotechnol..

[45] Razansky D., Distel M., Vinegoni C., Ma R., Perrimon N., Koster R.W., Ntziachristos V. (2009). Multispectral opto-acoustic tomography of deep-seated fluorescent proteins. in vivo. Nat. Photonics..

[46] Zhang E., Laufer J., Beard P. (2008). Backward-mode multiwavelength photoacoustic scanner using a planar Fabry-Perot polymer film ultrasound sensor for high-resolution three-dimensional imaging of biological tissues. Appl. Opt..

[47] Yang J.M., Favazza C., Chen R.M., Yao J.J., Cai X., Maslov K., Zhou Q.F., Shung K.K., Wang L.H.V. (2012). Simultaneous functional photoacoustic and ultrasonic endoscopy of internal organs. in vivo. Nat. Med..

[48] Paltauf G., Nuster R., Haltmeier M., Burgholzer P. (2007). Photoacoustic tomography using a Mach-Zehnder interferometer as an acoustic line detector. Appl. Opt..

[49] Kruger R.A., Lam R.B., Reinecke D.R., del Rio S.P., Doyle R.P. (2010). Photoacoustic angiography of the breast. Med. Phys..

[50] Manohar S., Kharine A., van Hespen J.C.G., Steenbergen W., van Leeuwen T.G. (2005). The twente photoacoustic mammoscope: System overview and performance. Phys. Med. Biol..

[51] Xia W.F., Piras D., van Hespen J.C.G., van Veldhoven S., Prins C., van Leeuwen T.G., Steenbergen W., Manohar S. (2013). An optimized ultrasound detector for photoacoustic breast tomography. Med. Phys..

[52] Huang S.-W., Chen S.-L., Ling T., Maxwell A., O'Donnell M., Guo L.J., Ashkenazi S. (2008). Low-noise wideband ultrasound detection using polymer microring resonators. Appl. Phys. Lett..

[53] Chen S.-L., Huang S.-W., Ling T., Ashkenazi S., Guo L. (2009). Polymer microring resonators for high-sensitivity and wideband photoacoustic imaging. IEEE Trans. Ultrason. Ferroelectr. Freq. Control..

[54] Rosenthal A., Razansky D., Ntziachristos V. (2011). High-sensitivity compact ultrasonic detector based on a pi-phase-shifted fiber Bragg grating. Opt. Lett..

[55] Oralkan O., Ergun A.S., Johnson J.A., Karaman M., Demirci U., Kaviani K., Lee T.H., Khuri-Yakub B.T. (2002). Capacitive micromachined ultrasonic transducers: Next-generation arrays for acoustic imaging?. IEEE Trans. Ultrason. Ferroelectr. Freq. Control..

[56] Caliano G., Carotenuto R., Cianci E., Foglietti V., Caronti A., Iula A., Pappalardo M. (2005). Design, fabrication and characterization of a capacitive micromachined ultrasonic probe for medical imaging. IEEE Trans. Ultrason. Ferroelectr. Freq. Control..

[57] Vaithilingam S., Ma T.-J., Furukawa Y., Wygant I.O., Xuefeng Z., de la Zerda A., Oralkan O., Kamaya A., Jeffrey R.B., Khuri-Yakub B.T. (2009). Three-dimensional photoacoustic imaging using a two-dimensional CMUT array. IEEE Trans. Ultrason. Ferroelectr. Freq. Control..

[58] Viator J.A., Choi B., Ambrose M., Spanier J., Nelson J.S. (2003). *In vivo* port-wine stain depth determination with a photoacoustic probe. Appl. Opt..

[59] Holan S.H., Viator J.A. (2008). Automated wavelet denoising of photoacoustic signals for circulating melanoma cell detection and burn image reconstruction. Phys. Med. Biol..

[60] Wang Y., Xing D., Zeng Y.G., Chen Q. (2004). Photoacoustic imaging with deconvolution algorithm. Phys. Med. Biol..

[61] Rosenthal A., Ntziachristos V., Razansky D. (2011). Optoacoustic methods for frequency calibration of ultrasonic sensors. IEEE Trans. Ultrason. Ferroelectr. Freq. Control..

[62] Norton S.J., Linzer M. (1981). Ultrasonic reflectivity imaging in three dimensions: Exact inverse scattering solutions for plane, cylindrical, and spherical apertures. IEEE Trans. Biomed. Eng..

[63] Kostli K.P., Frenz M., Bebie H., Weber H.P. (2001). Temporal backward projection of optoacoustic pressure transients using Fourier transform methods. Phys. Med. Biol..

[64] Kunyansky L.A. (2007). A series solution and a fast algorithm for the inversion of the spherical mean Radon transform. Inverse Probl..

[65] Xu Y., Feng D.Z., Wang L.H.V. (2002). Exact frequency-domain reconstruction for thermoacoustic tomography—I: Planar geometry. IEEE Trans. Med. Imaging..

[66] Xu Y., Xu M.H., Wang L.H.V. (2002). Exact frequency-domain reconstruction for thermoacoustic tomography—II: Cylindrical geometry. IEEE Trans. Med. Imaging..

[67] Kunyansky L. (2012). Fast reconstruction algorithms for the thermoacoustic tomography in certain domains with cylindrical or spherical symmetries. Inverse Probl. Imaging..

[68] Wang K., Anastasio M.A. (2012). A simple Fourier transform-based reconstruction formula for photoacoustic computed tomography with a circular or spherical measurement geometry. Phys. Med. Biol..

[69] Finch D., Rakesh P.S.K. (2004). Determining a function from its mean values over a family of spheres. Siam J. Math. Anal..

[70] Finch D., Rakesh H.M. (2007). Inversion of spherical means and the wave equation in even dimensions. Siam J. Appl. Math..

[71] Kunyansky L.A. (2007). Explicit inversion formulae for the spherical mean Radon transform. Inverse Probl..

[72] Xu M.H., Wang L.H.V. (2002). Time-domain reconstruction for thermoacoustic tomography in a spherical geometry. IEEE Trans. Med. Imaging..

[73] Xu M.H., Wang L.H.V. (2005). Universal back-projection algorithm for photoacoustic computed tomography. Phys. Rev. E.

[74] Xu Y., Wang L.H.V. (2004). Time reversal and its application to tomography with diffracting sources. Phys. Rev. Lett..

[75] Hristova Y., Kuchment P., Nguyen L. (2008). Reconstruction and time reversal in thermoacoustic tomography in acoustically homogeneous and inhomogeneous media. Inverse Probl..

[76] Treeby B.E., Zhang E.Z., Cox B.T. (2010). Photoacoustic tomography in absorbing acoustic media using time reversal. Inverse Probl..

[77] Cox B.T., Kara S., Arridge S.R., Beard P.C. (2007). k-space propagation models for acoustically heterogeneous media: Application to biomedical photoacoustics. J. Acoust. Soc. Am..

[78] Paltauf G., Viator J.A., Prahl S.A., Jacques S.L. (2002). Iterative reconstruction algorithm for optoacoustic imaging. J. Acoust. Soc. Am..

[79] Rosenthal A., Razansky D., Ntziachristos V. (2010). Fast semi-analytical model-based acoustic inversion for quantitative optoacoustic tomography. IEEE Trans. Med. Imaging..

[80] Wang K., Ermilov S.A., Su R., Brecht H.P., Oraevsky A.A., Anastasio M.A. (2011). An imaging model incorporating ultrasonic transducer properties for three-dimensional optoacoustic tomography. IEEE Trans. Med. Imaging..

[81] Yuan Z., Jiang H.B. (2006). Quantitative photoacoustic tomography: Recovery of optical absorption coefficient maps of heterogeneous media. Appl. Phys. Lett..

[82] Dean-Ben X., Buehler A., Ntziachristos V., Razansky D. (2012). Accurate model-based reconstruction algorithm for three-dimensional optoacoustic tomography. IEEE Trans. Med. Imaging.

[83] Yao L., Jiang H.B. (2009). Finite-element-based photoacoustic tomography in time domain. J. Opt. A Pure Appl. Opt..

[84] Golub G.H., van Loan C.F. (1996). Matrix Computations.

[85] Paige C.C., Saunders M.A. (1982). LSQR: An algorithm for sparse linear equations and sparse least squares. ACM Trans. Math. Softw..

[86] Buehler A., Deán-Ben X., Claussen J., Ntziachristos V., Razansky D. (2012). Three-dimensional optoacoustic tomography at video rate. Opt. Express..

[87] Wang K., Huang C., Kao Y.-J., Chou C.-Y., Oraevsky A.A., Anastasio M.A. (2013). Accelerating image reconstruction in three-dimensional optoacoustic tomography on graphics processing units. Med. Phys..

[88] Pratx G., Xing L. (2011). GPU computing in medical physics: A review. Med. Phys..

[89] Rosenthal A., Jetzfellner T., Razansky D., Ntziachristos V. (2012). Efficient framework for model-based tomographic image reconstruction using wavelet packets. IEEE Trans. Med. Imaging..

[90] Razansky D., Buehler A., Ntziachristos V. (2011). Volumetric real-time multispectral optoacoustic tomography of biomarkers. Nat. Protoc..

[91] Gamelin J., Maurudis A., Aguirre A., Huang F., Guo P.Y., Wang L.V., Zhu Q. (2009). A real-time photoacoustic tomography system for small animals. Opt. Express..

[92] Taruttis A., Herzog E., Razansky D., Ntziachristos V. (2010). Real-time imaging of cardiovascular dynamics and circulating gold nanorods with multispectral optoacoustic tomography. Opt. Express..

[93] Li M.L., Zhang H.F., Maslov K., Stoica G., Wang L.H.V. (2006). Improved *in vivo* photoacoustic microscopy based on a virtual-detector concept. Opt. Lett..

[94] Yang X.M., Li M.L., Wang L.H.V. (2007). Ring-based ultrasonic virtual point detector with applications to photoacoustic tomography. Appl. Phys. Lett..

[95] Li C.H., Wang L.V. (2008). High-numerical-aperture-based virtual point detectors for photoacoustic tomography. Appl. Phys. Lett..

[96] Burgholzer P., Hofer C., Paltauf G., Haltmeier M., Scherzer O. (2005). Thermoacoustic tomography with integrating area and line detectors. IEEE Trans. Ultrason. Ferroelectr. Freq. Control.

[97] Burgholzer P., Bauer-Marschallinger J., Grun H., Haltmeier M., Paltauf G. (2007). Temporal back-projection algorithms for photoacoustic tomography with integrating line detectors. Inverse Probl..

[98] Haltmeier M., Scherzer O., Burgholzer P., Paltauf G. (2004). Thermoacoustic computed tomography with large planar receivers. Inverse Probl..

[99] Gratt S., Passler K., Nuster R., Paltauf G. (2011). Photoacoustic section imaging with an integrating cylindrical detector. Biomed. Opt. Express..

[100] Nuster R., Zangerl G., Haltmeier M., Paltauf G. (2010). Full field detection in photoacoustic tomography. Opt. Express..

[101] Rosenthal A., Ntziachristos V., Razansky D. (2011). Model-based optoacoustic inversion with arbitrary-shape detectors. Med. Phys..

[102] Duck F.A. (1990). Physical Properties of Tissue: A Comprehensive Reference Book.

[103] Manohar S., Willemink R.G.H., van der Heijden F., Slump C.H., van Leeuwen T.G. (2007). Concomitant speed-of-sound tomography in photoacoustic imaging. Appl. Phys. Lett..

[104] Jose J., Willemink R.G.H., Resink S., Piras D., van Hespen J.C.G., Slump C.H., Steenbergen W., van Leeuwen T.G., Manohar S. (2011). Passive element enriched photoacoustic computed tomography (PER PACT) for simultaneous imaging of acoustic propagation properties and light absorption. Opt. Express..

[105] Treeby B.E., Varslot T.K., Zhang E.Z., Laufer J.G., Beard P.C. (2011). Automatic sound speed selection in photoacoustic image reconstruction using an autofocus approach. J. Biomed. Opt..

[106] Yuan Z., Jiang H.B. (2007). Three-dimensional finite-element-based photoacoustic tomography: Reconstruction algorithm and simulations. Med. Phys..

[107] Agranovsky M., Kuchment P. (2007). Uniqueness of reconstruction and an inversion procedure for thermoacoustic and photoacoustic tomography with variable sound speed. Inverse Probl..

[108] Modgil D., Anastasio M.A., La Riviere P.J. (2010). Image reconstruction in photoacoustic tomography with variable speed of sound using a higher-order geometrical acoustics approximation. J. Biomed. Opt..

[109] Xu M.H., Wang L.V. (2003). Analytic explanation of spatial resolution related to bandwidth and detector aperture size in thermoacoustic or photoacoustic reconstruction. Phys. Rev. E.

[110] La Riviere P.J., Zhang J., Anastasio M.A. (2006). Image reconstruction in optoacoustic tomography for dispersive acoustic media. Opt. Lett..

[111] Wang L.H.V., Yang X.M. (2007). Boundary conditions in photoacoustic tomography and image reconstruction. J. Biomed. Opt..

[112] Anastasio M.A., Zhang J., Pan X.C., Zou Y., Ku G., Wang L.H.V. (2005). Half-time image reconstruction in thermoacoustic tomography. IEEE Trans. Med. Imaging..

[113] Dean-Ben X.L., Ma R., Razansky D., Ntziachristos V. (2011). Statistical approach for optoacoustic image reconstruction in the presence of strong acoustic heterogeneities. IEEE Trans. Med. Imaging..

[114] Dean-Ben X.L., Ntziachristos V., Razansky D. (2012). Artefact reduction in optoacoustic tomographic imaging by estimating the distribution of acoustic scatterers. J. Biomed. Opt..

[115] Xu Y., Wang L.V., Ambartsoumian G., Kuchment P. (2004). Reconstructions in limited-view thermoacoustic tomography. Med. Phys..

[116] Buehler A., Rosenthal A., Jetzfellner T., Dima A., Razansky D., Ntziachristos V. (2011). Model-based optoacoustic inversions with incomplete projection data. Med. Phys..

[117] Yao L., Jiang H.B. (2011). Photoacoustic image reconstruction from few-detector and limited-angle data. Biomed. Opt. Express..

[118] Paltauf G., Nuster R., Burgholzer P. (2009). Weight factors for limited angle photoacoustic tomography. Phys. Med. Biol..

[119] Kunyansky L.A. (2008). Thermoacoustic tomography with detectors on an open curve: An efficient reconstruction algorithm. Inverse Probl..

[120] Haltmeier M., Scherzer O., Zangerl G. (2009). A reconstruction algorithm for photoacoustic imaging based on the nonuniform FFT. IEEE Trans. Med. Imaging..

[121] Donoho D.L. (2006). Compressed sensing. IEEE Trans. Inf. Theory..

[122] Candes E.J., Wakin M.B. (2008). An introduction to compressive sampling. IEEE Signal Process. Mag..

[123] Lustig M., Donoho D., Pauly J.M. (2007). Sparse MRI: The application of compressed sensing for rapid MR imaging. Magn. Reson. Med..

[124] Provost J., Lesage F. (2009). The application of compressed sensing for photo-acoustic tomography. IEEE Trans. Med. Imaging..

[125] Guo Z.J., Li C.H., Song L.A., Wang L.H.V. (2010). Compressed sensing in photoacoustic tomography *in vivo*. J. Biomed. Opt..

[126] Meng J., Wang L.H.V., Liang D., Song L. (2012). *In vivo* optical-resolution photoacoustic computed tomography with compressed sensing. Opt. Lett..

[127] Wang L., Jacques S.L., Zheng L. (1995). MCML-Monte Carlo modeling of light transport in multi-layered tissues. Comput. Methods Programs Biomed..

[128] Bu S.H., Liu Z.B., Shiina T., Kondo K., Yamakawa M., Fukutani K., Someda Y., Asao Y. (2012). Model-based reconstruction integrated with fluence compensation for photoacoustic tomography. IEEE Trans. Biomed. Eng..

[129] Zhu C., Liu Q. (2013). Review of monte carlo modeling of light transport in tissues. J. Biomed. Opt..

[130] Arridge S.R., Schweiger M., Hiraoka M., Delpy D.T. (1993). A finite element approach for modeling photon transport in tissue. Med. Phys..

[131] Arridge S.R. (1999). Optical tomography in medical imaging. Inverse Probl..

[132] Tarvainen T., Vauhkonen M., Kolehmainen V., Arridge S.R., Kaipio J.P. (2005). Coupled radiative transfer equation and diffusion approximation model for photon migration in turbid medium with low-scattering and non-scattering regions. Phys. Med. Biol..

[133] Paulsen K.D., Jiang H. (1995). Spatially varying optical property reconstruction using a finite element diffusion equation approximation. Med. Phys..

[134] Kienle A., Patterson M.S. (1997). Improved solutions of the steady-state and the time-resolved diffusion equations for reflectance from a semi-infinite turbid medium. J. Opt. Soc. Am. A Opt. Image Sci. Vis..

[135] Cox B.T., Arridge S.R., Kostli K.P., Beard P.C. (2005). Quantitative Photoacoustic imaging: Fitting a model of light transport to the initial pressure distribution. Proc. SPIE..

[136] Cox B.T., Arridge S.R., Kostli K.P., Beard P.C. (2006). Two-dimensional quantitative photoacoustic image reconstruction of absorption distributions in scattering media by use of a simple iterative method. Appl. Opt..

[137] Maslov K., Zhang H.F., Hu S., Wang L.V. (2008). Optical-resolution photoacoustic microscopy for *in vivo* imaging of single capillaries. Opt. Lett..

[138] Jetzfellner T., Razansky D., Rosenthal A., Schulz R., Englmeier K.H., Ntziachristos V. (2009). Performance of iterative optoacoustic tomography with experimental data. Appl. Phys. Lett..

[139] Shao P., Cox B., Zemp R.J. (2011). Estimating optical absorption, scattering, and Grueneisen distributions with multiple-illumination photoacoustic tomography. Appl. Opt..

[140] Laufer J., Cox B., Zhang E., Beard P. (2010). Quantitative determination of chromophore concentrations from 2D photoacoustic images using a nonlinear model-based inversion scheme. Appl. Opt..

[141] Bal G., Uhlmann G. (2010). Inverse diffusion theory of photoacoustics. Inverse Probl..

[142] Shao P., Harrison T., Zemp R.J. (2012). Iterative algorithm for multiple illumination photoacoustic tomography (MIPAT) using ultrasound channel data. Biomed. Opt. Express..

[143] Rosenthal A., Razansky D., Ntziachristos V. (2009). Quantitative optoacoustic signal extraction using sparse signal representation. IEEE Trans. Med. Imaging..

[144] Cox B.T., Laufer J.G., Beard P.C. (2010). Quantitative photoacoustic image reconstruction using fluence dependent chromophores. Biomed. Opt. Express..

[145] Yin L., Wang Q., Zhang Q.Z., Jiang H.B. (2007). Tomographic imaging of absolute optical absorption coefficient in turbid media using combined photoacoustic and diffusing light measurements. Opt. Lett..

[146] Li X.Q., Xi L., Jiang R.X., Yao L., Jiang H.B. (2011). Integrated diffuse optical tomography and photoacoustic tomography: Phantom validations. Biomed. Opt. Express..

[147] Bauer A.Q., Nothdurft R.E., Erpelding T.N., Wang L.H.V., Culver J.P. (2011). Quantitative photoacoustic imaging: Correcting for heterogeneous light fluence distributions using diffuse optical tomography. J. Biomed. Opt..

[148] Maslov K., Zhang H.F., Wang L.V. (2007). Effects of wavelength-dependent fluence attenuation on the noninvasive photoacoustic imaging of hemoglobin oxygen saturation in subcutaneous vasculature. in vivo. Inverse Probl..

[149] Kostli K.P., Frenz M., Weber H.P., Paltauf G., Schmidt-Kloiber H. (2000). Optoacoustic infrared spectroscopy of soft tissue. J. Appl. Phys..

[150] Laufer J., Delpy D., Elwell C., Beard P. (2007). Quantitative spatially resolved measurement of tissue chromophore concentrations using photoacoustic spectroscopy: Application to the measurement of blood oxygenation and haemoglobin concentration. Phys. Med. Biol..

[151] Sethuraman S., Amirian J.H., Litovsky S.H., Smalling R.W., Emelianov S.Y. (2008). Spectroscopic intravascular photoacoustic imaging to differentiate atherosclerotic plaques. Opt. Express..

[152] Razansky D. (2012). Multispectral optoacoustic tomography-volumetric color hearing in real time. IEEE J. Sel. Top. Quantum Electron..

[153] Van Veen R.L.P., Sterenborg H.J., Pifferi A., Torricelli A., Cubeddu R. (2004). Determination of VIS-NIR Absorption Coefficients of Mammalian Fat, with Time- and Spatially Resolved Diffuse Reflectance and Transmission Spectroscopy.

[154] Ashkenazi S., Huang S.W., Horvath T., Koo Y.E.L., Kopelman R., Kopelman R. (2008). Photoacoustic probing of fluorophore excited state lifetime with application to oxygen sensing. J. Biomed. Opt..

[155] Huang S.W., Eary J.F., Jia C.X., Huang L.Y., Ashkenazi S., O'Donnell M. (2009). Differential-absorption photoacoustic imaging. Opt. Lett..

[156] Glatz J., Deliolanis N.C., Buehler A., Razansky D., Ntziachristos V. (2011). Blind source unmixing in multi-spectral optoacoustic tomography. Opt. Express..

[157] Joliffe I.T. (2002). Principal Component Analysis.

[158] Hyvarinen A., Oja E. (2000). Independent component analysis: Algorithms and applications. Neural Netw..

[159] Maslov K., Stoica G., Wang L.H.V. (2005). *In vivo* dark-field reflection-mode photoacoustic microscopy. Opt. Lett.

[160] Taruttis A., Wildgruber M., Kosanke K., Beziere N., Licha K., Haag R., Aichler M., Walch A., Rummeny E., Ntziachristos V. (2013). Multispectral optoacoustic tomography of myocardial infarction. Photoacoustics..

[161] Buehler A., Herzog E., Ale A., Smith B.D., Ntziachristos V., Razansky D. (2012). High resolution tumor targeting in living mice by means of multispectral optoacoustic tomography. EJNMMI Res..

[162] Smith B.A., Akers W.J., Leevy W.M., Lampkins A.J., Xiao S.Z., Wolter W., Suckow M.A., Achilefu S., Smith B.D. (2010). Optical imaging of mammary and prostate tumors in living animals using a synthetic near infrared Zinc(II)-dipicolylamine probe for anionic cell surfaces. J. Am. Chem. Soc..

[163] Xiao J.Y., Yao L., Sun Y., Sobel E.S., He J.S., Jiang H.B. (2010). Quantitative two-dimensional photoacoustic tomography of osteoarthritis in the finger joints. Opt. Express..

[164] Razansky D., Harlaar N.J., Hillebrands J.L., Taruttis A., Herzog E., Zeebregts C.J., van Dam G.M., Ntziachristos V. (2012). Multispectral optoacoustic tomography of matrix metalloproteinase activity in a vulnerable human carotid plaque. Mol. Imaging Biol..

[165] Nissen S.E., Yock P. (2001). Intravascular ultrasound-novel pathophysiological insights and current clinical applications. Circulation.

[166] Razansky R.N., Rosenthal A., Mallas G., Razansky D., Jaffer F.A., Ntziachristos V. (2010). Near-infrared fluorescence catheter system for two-dimensional intravascular imaging. in vivo. Opt. Express.

[167] Sethuraman S., Aglyamov S.R., Amirian J.H., Smalling R.W., Emelianov S.Y. (2007). Intravascular photoacoustic imaging using an IVUS imaging catheter. IEEE Trans. Ultrason. Ferroelectr. Freq. Control.

[168] Yang J.M., Maslov K., Yang H.C., Zhou Q.F., Shung K.K., Wang L.H.V. (2009). Photoacoustic endoscopy. Opt. Lett..

[169] Jansen K., van der Steen A.F.W., van Beusekom H.M.M., Oosterhuis J.W., van Soest G. (2011). Intravascular photoacoustic imaging of human coronary atherosclerosis. Opt. Lett..

[170] ANSI Standard Z136 (2000). 1-2000: For Safe Use of Lasers.

[171] Deán-Ben X.L., Razansky D., Ntziachristos V. (2011). The effects of acoustic attenuation in optoacoustic signals. Phys. Med. Biol..

[172] Razansky D., Baeten J., Ntziachristos V. (2009). Sensitivity of molecular target detection by multispectral optoacoustic tomography (MSOT). Med. Phys..

[173] Ku G., Wang X., Xie X., Stoica G., Wang L.V. (2005). Imaging of tumor angiogenesis in rat brains *in vivo* by photoacoustic tomography. Appl. Opt..

[174] Stein E.W., Maslov K., Wang L.V. (2009). Noninvasive, *in vivo* imaging of blood-oxygenation dynamics within the mouse brain using photoacoustic microscopy. J. Biomed. Opt..

[175] Fainchtein R., Stoyanov B.J., Murphy J.C., Wilson D.A., Hanley D.F. (2000). Local Determination of Hemoglobin Concentration and Degree of Oxygenation in Tissue by Pulsed Photoacoustic Spectroscopy. Proc. SPIE..

[176] Laufer J., Elwell C., Delpy D., Beard P. (2005). *In vitro* measurements of absolute blood oxygen saturation using pulsed near-infrared photoacoustic spectroscopy: Accuracy and resolution. Phys. Med. Biol..

[177] Zhang H.F., Maslov K., Sivaramakrishnan M., Stoica G., Wang L.V. (2007). Imaging of hemoglobin oxygen saturation variations in single vessels *in vivo* using photoacoustic microscopy. Appl. Phys. Lett..

